# Antiallergic Activity of 3-*O*-Dodecyl-l-ascorbic Acid

**DOI:** 10.3390/molecules29010069

**Published:** 2023-12-21

**Authors:** Takeru Koga, Naoaki Kawahara, Mei Aburada, Asako Ono, Shiori Mae, Aina Yoshida, Yuji Iwaoka, Hideyuki Ito, Akihiro Tai

**Affiliations:** 1 Graduate School of Technology, Industrial and Social Sciences, Tokushima University, 2-1 Minamijosanjima-Cho, Tokushima 770-8513, Japan; koga.takeru@tokushima-u.ac.jp; 2 Graduate School of Sciences and Technology for Innovation, Tokushima University, 2-1 Minamijosanjima-Cho, Tokushima 770-8513, Japan; 3 Faculty of Life and Environmental Sciences, Prefectural University of Hiroshima, 5562 Nanatsuka-Cho, Shobara 727-0023, Japan; 4 Faculty of Health and Welfare Science, Okayama Prefectural University, 111 Kuboki, Soja, Okayama 719-1197, Japan; iwaoka@fhw.oka-pu.ac.jp (Y.I.); hito@fhw.oka-pu.ac.jp (H.I.)

**Keywords:** ascorbic acid derivatives, degranulation inhibitory activity, passive cutaneous anaphylaxis (PCA) reaction, RBL-2H3 cells

## Abstract

2-*O*-Alkyl-l-ascorbic acids and 3-*O*-alkyl-l-ascorbic acids were synthesized, and their degranulation inhibitory activities were evaluated. Among ascorbic acid derivatives with butyl, octyl, dodecyl, hexadecyl, and octadecyl groups introduced at the C-2 or C-3 positions, an AA derivative with a dodecyl group introduced at the C-3 position, 3-*O*-dodecyl-l-ascorbic acid (compound **8**), showed the strongest inhibitory activity against antigen-stimulated degranulation. Compound **8** also inhibited calcium ionophore-stimulated degranulation. Compound **11**, in which the hydroxyl group at the C-6 position of compound **8** was substituted with an amino group, and compound **12**, in which the dodecyloxy group at the C-3 position of compound **8** was exchanged with a dodecylamino group, were synthesized, and these derivatives showed weaker inhibitory activity against antigen-stimulated degranulation than that of compound **8**. In addition, orally administered compound **8** inhibited passive cutaneous anaphylaxis reactions in mice with a potency equal to that of oxatomide, an antiallergic agent. These results suggest that compound **8** may be a candidate for antiallergic treatment.

## 1. Introduction

Allergic disease is one of the most prevalent diseases in the world [1,2]. The burden of allergic disease is continuing to increase and is a problem to be solved. There are four main types of allergies, and type I allergy is associated with major allergic symptoms such as asthma and anaphylaxis [3]. In the type I allergic reaction, immunoglobulin E (IgE) binds to Fcε receptor I expressed on the membrane of mast cells, and an antigen called an allergen such as pollen crosslinks IgE. This induces the release of chemical mediators such as histamine stored in mast cells to the outside of the cells. The phenomenon of the release of an intracellular chemical to the outside of the cell is called degranulation. The released histamine causes increased vascular permeability and smooth muscle contraction, leading to allergic symptoms [4,5]. Hence, it has been considered that suppression of the degranulation of chemical mediators from mast cells should lead to improvement and prevention of allergic symptoms.

L-Ascorbic acid (AA) is a water-soluble compound known as vitamin C. AA is contained in foods and is also used in cosmetics since it has a potent antioxidant activity [6,7]. The enediol structure of AA, which is composed of hydroxyl groups at the C-2 and C-3 positions, is easily oxidized and is extremely unstable in an aqueous solution [8]. As a stable lipophilic AA derivative with a C16 straight-acyl chain introduced at the C-6 position of AA and a glucose molecule introduced at the C-2 position, 2-*O*-D-glucopyranosyl-6-*O*-hexadecanoyl-l-ascorbic acid (6-Palm-AA-2G) has been developed [9]. 6-Palm-AA-2G is a provitamin C agent that is poorly oxidized and can be intravitally metabolized by esterase and α-glucosidase to provide AA [10]. A previous study revealed that 6-Palm-AA-2G showed degranulation inhibitory activity as an effect of the derivative itself [11]. Hence, in order to clarify the essential structure of 6-Palm-AA-2G for its degranulation inhibitory activity and to develop ascorbic acid derivatives with excellent antiallergic activity, 6-*O*-hexadecanoyl-l-ascorbic acid derivatives were synthesized and their structure–activity relationships in degranulation inhibitory activity were investigated [12]. Based on the results, 6-deoxy-2-*O*-methyl-6-(*N*-hexadecanoyl)amino-l-ascorbic acid (2-Me-6-*N*-Palm-AA) was developed as a degranulation inhibitor with greater potency than that of 6-Palm-AA-2G. Percutaneous administration of 2-Me-6-*N*-Palm-AA also inhibited passive cutaneous anaphylaxis (PCA) reactions in mice, indicating that 2-Me-6-*N*-Palm-AA has antiallergic activity.

Previously, 2-*O*-alkyl-l-ascorbic acids (2-*O*-AlkylAA) and 3-*O*-alkyl-l-ascorbic acids (3-*O*-AlkylAA), which have straight-chain alkyl groups introduced at the C-2 or C-3 positions of AA, were synthesized [13,14]. 2-*O*-Octadecyl-l-ascorbic acid (2-*O*-OctadecylAA), an AA derivative with an octadecyl group introduced at the C-2 position of AA, significantly suppressed the development of hepatocellular carcinomas in mice [15] and inhibited ischemic reperfusion injury [16,17]. 3-*O*-Ethyl-l-ascorbic acid (3-*O*-EthylAA), an AA derivative with an ethyl group introduced at the C-3 position of AA, exhibited neurite outgrowth-promoting activity [18]. In addition, 3-*O*-EthylAA showed tyrosinase inhibitory activity [19] and has been applied as a material for cosmetics. On the other hand, it has been reported that 2-*O*-OctadecylAA does not exhibit anti-scurvy activity, which is an AA activity [20]. These facts suggest that 2-*O*-AlkylAA and 3-*O*-AlkylAA are monosubstituted AA derivatives that exert their activity as derivatives themselves as opposed to being metabolized in vivo to exert their vitamin C activity.

In this study, AA derivatives with butyl, octyl, dodecyl, hexadecyl, or octadecyl groups introduced at the C-2 or C-3 positions were synthesized, and their degranulation inhibitory activities were evaluated. Then, an AA derivative with the hydroxyl group at the C-6 position substituted with an amino group and an AA derivative with the dodecyloxy group at the C-3 position exchanged with a dodecylamino group using 3-*O*-DodecylAA, which showed the strongest degranulation inhibitory activity, were synthesized, and their degranulation inhibitory activities were compared. The inhibitory activity of orally administered 3-*O*-DodecylAA against PCA reaction in mice was also investigated.

## 2. Results and Discussion

**Figure 1:**
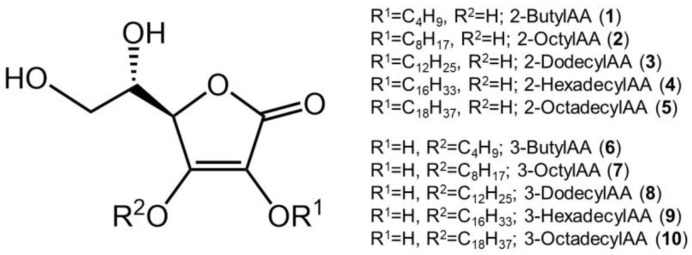
Chemical structures of 2-*O*-alkylascorbic acids (2-*O*-AlkylAA) and 3-*O*-alkylascorbic acids (3-*O*-AlkylAA).

**Figure 2:**
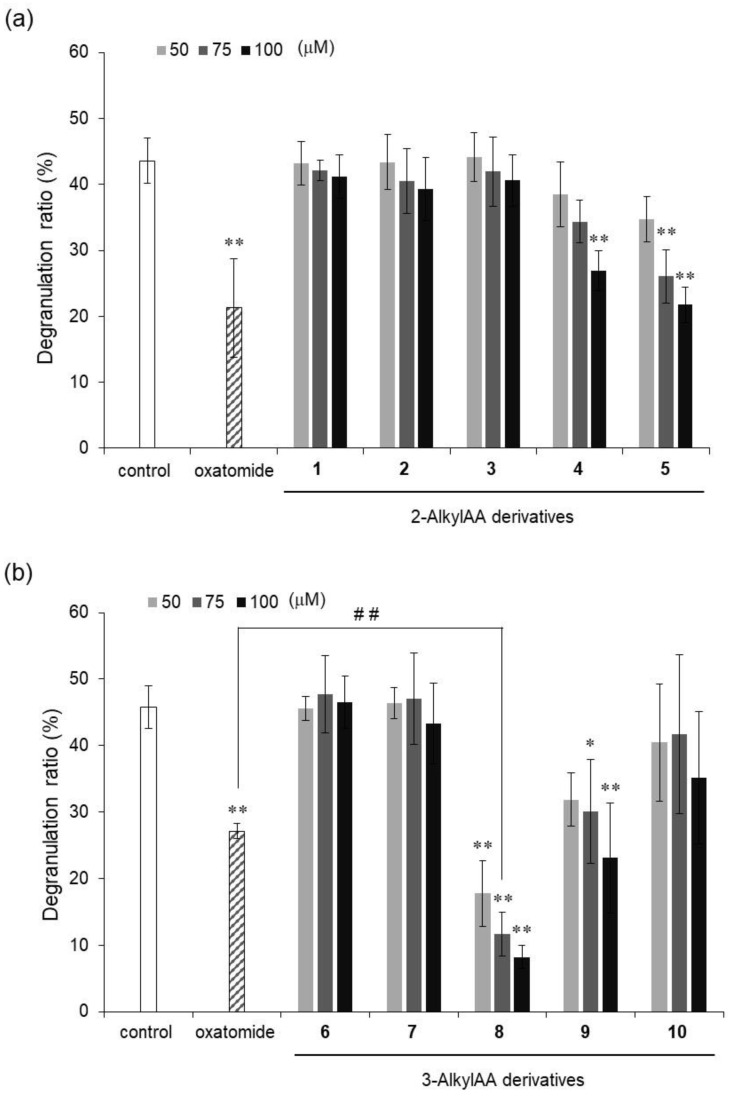
Inhibitory activities of 2-*O*-AlkylAA (**a**) and 3-*O*-AlkylAA (**b**) against antigen-stimulated degranulation in RBL-2H3 cells. Oxatomide (75 μM) was used as a positive control. Anti-dinitrophenyl (DNP)-immunoglobulin E-sensitized RBL-2H3 cells were incubated with the indicated AA derivatives and stimulated with DNP-human serum albumin. All data represent means ± SD of three independent experiments. * *p* < 0.05 and ** *p* < 0.01 (Dunnett’s test) as compared with the control. ## *p* < 0.01 (*t*-test).

To investigate whether monosubstituted AA derivatives exhibit the same degranulation inhibitory activity as that of disubstituted AA derivatives, 6-Palm-AA-2G and 2-Me-6-*N*-Palm-AA, various 2-*O*-AlkylAA and 3-*O*-AlkylAA derivatives were synthesized. 2-*O*-Butyl-l-ascorbic acid (**1**), 2-*O*-octyl-l-ascorbic acid (**2**), 2-*O*-dodecyl-l-ascorbic acid (**3**), 2-*O*-hexadecyl-l-ascorbic acid (**4**), and 2-*O*-octadecyl-l-ascorbic acid (**5**) were prepared as 2-*O*-AlkylAA derivatives; 3-*O*-butyl-l-ascorbic acid (**6**), 3-*O*-octyl-l-ascorbic acid (**7**), 3-*O*-dodecyl-l-ascorbic acid (**8**), 3-*O*-hexadecyl-l-ascorbic acid (**9**), and 3-*O*-octadecyl-l-ascorbic acid (**10**) were prepared as 3-*O*-AlkylAA derivatives (Figure 1). First, the antigen-stimulated degranulation inhibitory activities of the synthesized 2-*O*-AlkylAA and 3-*O*-AlkylAA derivatives in RBL-2H3 cells were evaluated (Figure 2).

The degranulation reaction releases chemical mediators such as histamine and leukotrienes, which cause various allergic reactions. Thus, degranulation inhibitors can be candidates of drugs for suppressing allergic symptoms. The antigen-stimulated degranulation inhibitory activity of each of these compounds was compared with that of oxatomide, which is known to have degranulation inhibitory activity [21,22] and is used for treatment of allergies. Among the 2-*O*-AlkylAA derivatives (compounds **1**–**5**), compounds **4** and **5** showed significant degranulation inhibitory activity, while compounds **1**, **2**, and **3** did not show inhibitory activity. Among the 3-*O*-AlkylAA derivatives (compounds **6**–**10**), compounds **8** and **9** showed significant degranulation inhibitory activity, while compounds **6**, **7**, and **10** did not show inhibitory activity. These results suggest that degranulation inhibitory activity became stronger as the alkyl chain of the derivative became longer in 2-*O*-AlkylAA derivatives. These results also showed that a certain degree of alkyl chain length was important for inhibitory activity in 3-*O*-AlkylAA derivatives. Compound **8** showed the strongest activity of all the evaluated 2-*O*-AlkylAA and 3-*O*-AlkylAA derivatives, and the intensity of its activity exceeded that of oxatomide. On the other hand, compounds **4**, **5**, and **9** showed the same degree of inhibitory activity as that of oxatomide or weaker inhibitory activity than that of oxatomide. These results indicated that the monosubstituted AA derivatives, as well as the disubstituted AA derivatives, have potent antigen-stimulated degranulation inhibitory activity and that compound **8** has the strongest activity of all of the evaluated derivatives.

**Figure 3:**
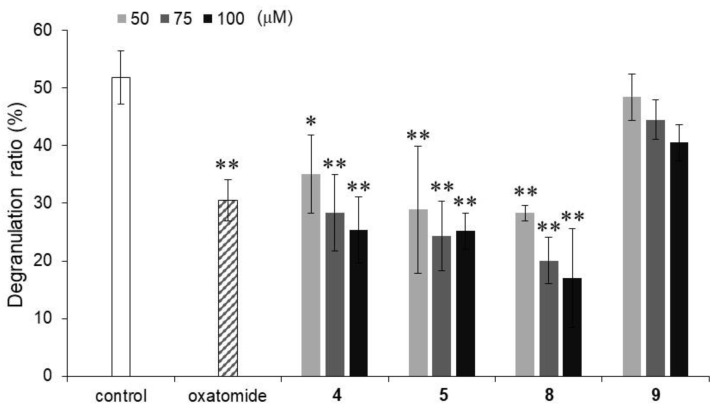
Inhibitory activities of compounds **4**, **5**, **8**, and **9** against calcium ionophore A23187-stimulated degranulation in RBL-2H3 cells. Oxatomide (75 μM) was used as a positive control. All data represent means ± SD of three independent experiments. * *p* < 0.05 and ** *p* < 0.01 (Dunnett’s test) compared with the control.

Furthermore, the inhibitory activities against calcium ionophore A23187-stimulated degranulation were evaluated for compounds **4**, **5**, **8**, and **9**, which significantly inhibited antigen-stimulated degranulation (Figure 3). Calcium signaling is one of the downstream signaling pathways activated by antigen stimulation [23]. The calcium ionophore-stimulated degranulation inhibitory activities of compounds **4**, **5**, and **8** were comparable to the inhibitory activity of oxatomide, while compound **9** did not show inhibitory activity. These results suggested that compounds **4**, **5**, and **8** elicit inhibitory activity downstream of calcium signaling and that compound **9** shows a degranulation inhibitory activity with a different mechanism from that of the inhibitory activities of compounds **4**, **5**, and **8**. Among the four alkylated AA derivatives that have inhibitory activity, compound **8** showed relatively strong inhibitory activity against degranulation stimulated by the calcium ionophore. In our previous study, 6-Palm-AA-2G did not inhibit calcium ionophore-stimulated degranulation, while 2-Me-6-*N*-Palm-AA showed strong inhibitory activity at 60 μM [12]. Compound **8** was found to be the strongest inhibitor of antigen-stimulated or calcium ionophore-stimulated degranulation responses, although the intensity of the inhibitory activity of compound **8** against calcium ionophore-stimulated degranulation was not as strong as that of 2-Me-6-*N*-Palm-AA. Therefore, compound **8** appeared to be a new candidate for an excellent degranulation inhibitor since it is a simple monosubstituted stable AA derivative with strong inhibitory activity against degranulation stimulated by an antigen and a calcium ionophore.

**Figure 4:**
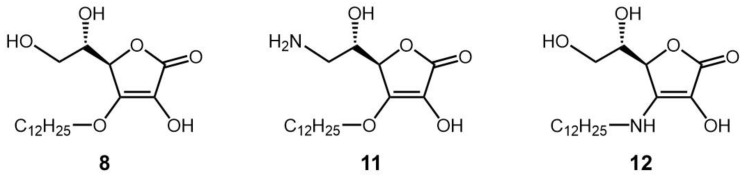
Chemical structures of derivatives of compound **8** (compounds **11** and **12**).

**Figure 5:**
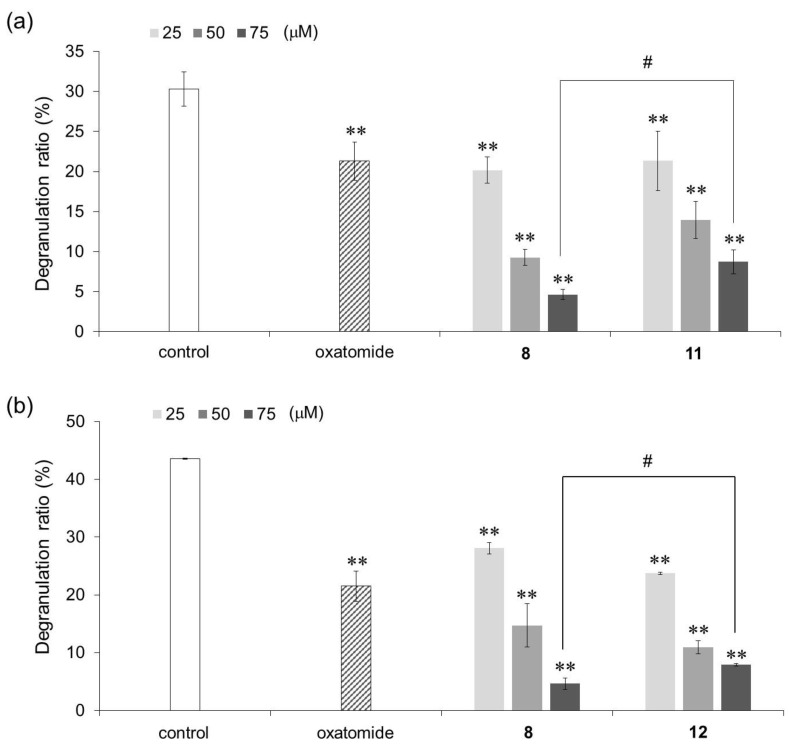
Inhibitory activities of compounds **11** (**a**) and **12** (**b**) against antigen-stimulated degranulation in RBL-2H3 cells. Oxatomide (75 μM) was used as a positive control. Anti-dinitrophenyl (DNP)-immunoglobulin E-sensitized RBL-2H3 cells were incubated with the indicated AA derivatives and stimulated with DNP-human serum albumin. All data represent means ± SD of three independent experiments. ** *p* < 0.01 (Dunnett’s test) as compared with the control. # *p* < 0.05 (*t*-test).

Next, an AA derivative with the hydroxyl group at the C-6 position of compound **8** substituted with an amino group, 6-deoxy-6-amino-3-*O*-dodecyl-l-ascorbic acid (compound **11**), and an AA derivative with the dodecyloxy group at the C-3 position of compound **8** exchanged with a dodecylamino group, 3-deoxy-3-dodecylamino-l-ascorbic acid (compound **12**), were synthesized, and their antigen-stimulated degranulation inhibitory activities were compared. The chemical structures of compounds **11** and **12** are shown in Figure 4. It was found that the degranulation inhibitory activity of compound **11** at 75 μM was weaker than that of compound **8** (Figure 5a). Also, the degranulation inhibitory activity of compound **12** at 75 μM was weaker than that of compound **8** (Figure 5b). These results showed that compound **8** was the most potent inhibitor against antigen-stimulated degranulation. Furthermore, compound **8** could be easily synthesized in a one-step reaction and the yield of compound **8** (40.0%) was much higher than the yields of compounds **11** (3.3%) and **12** (2.5%). Therefore, compound **8** is the most suitable compound for application as an antiallergic agent among the AA derivatives evaluated in this study.

**Figure 6:**
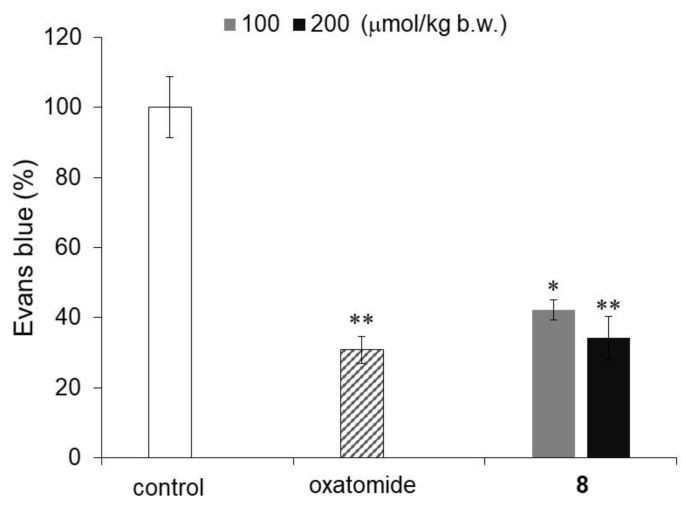
Inhibitory activity of compound **8** against the antigen-stimulated PCA reaction in mice. Mice were orally administered the indicated samples: control (*n* = 6), oxatomide at a dose of 100 μmol/kg b.w. (*n* = 5), and compound **8** at doses of 100 μmol/kg b.w. (*n* = 5) and 200 μmol/kg b.w. (*n* = 6). All data represent the means ± SE. * *p* < 0.05, ** *p* < 0.01 (Dunnett’s test) compared with the control.

Finally, an IgE-mediated passive cutaneous anaphylaxis (PCA) reaction test in mice was performed to investigate the antiallergic activity of compound **8**, which showed the strongest degranulation inhibitory activity among the monosubstituted AA derivatives evaluated in this study. The PCA response is a model experiment for the inflammatory response of a type I allergy in animals, and the PCA response in the mouse ear has been used to investigate various antiallergic actions. The ears of mice are sensitized with IgE antibodies, and when an antigen and dye are intravenously injected, a PCA response is induced to increase vascular permeability. The antiallergic activity of compound **8** was evaluated by measuring the amount of dye that leaked from the vessels. Oxatomide and compound **8** were administered orally to mice 2 h before antigen administration. Compound **8** and oxatomide significantly inhibited the PCA response (Figure 6). The intensity of the inhibitory activity of compound **8** against the PCA reaction was similar to that of oxatomide, an antiallergic agent that is used for the treatment of allergic diseases. Previously, it was reported that 6-Palm-AA-2G and 2-Me-6-*N*-Palm-AA inhibited the PCA reaction in mice [11,12]. 6-Palm-AA-2G, which has been shown to be a degranulation inhibitor, is easily metabolized to AA in vivo [10,11]. In addition, 2-Me-6-*N*-Palm-AA, which has strong degranulation inhibitory activity, has been found to be partially hydrolyzed at the cellular level [12]. An experiment using a skin model showed that some of the 6-Acyl-AA derivatives penetrate the skin in an intact form and that some of them are hydrolyzed [11]. Hence, the PCA response inhibitory activities of 6-Palm-AA-2G and 2-Me-6-*N*-Palm-AA were evaluated by percutaneous administration to the ears of mice. Therefore, compound **8** was the first AA derivative to inhibit the PCA reaction by oral administration to mice.

In this study, to find simple monosubstituted AA derivatives with antiallergic activity, 2-*O*-AlkylAA and 3-*O*-AlkylAA derivatives were synthesized and their inhibitory activities against antigen-stimulated degranulation were evaluated. Among the evaluated monosubstituted AA derivatives, compound **8**, which has a dodecyl group at the C-3 position of AA, showed the strongest degranulation inhibitory activity. Compound **8** also exhibited inhibitory activity against calcium ionophore-stimulated degranulation. A comparison of the antigen-stimulated degranulation inhibitory activities of compound **8** and its analogues, compounds **11** and **12**, showed that compound **8** has the strongest inhibitory activity. Therefore, compound **8** is considered to be the most suitable antiallergic candidate among the AA derivatives. In addition, compound **8** inhibited PCA reactions via oral administration in mice with potency equal to that of oxatomide, which is used for antiallergic treatment. This study showed that compound **8** is an excellent monosubstituted AA derivative with antiallergic activity. Compound **8** is thus a new effective candidate of drugs for treatment of type I allergies.

## 3. Materials and Methods

### 3.1. Chemicals

1-Iodobutane, 1-iodooctane, 1-iodododecane, 1-iodohexadecane, and 1-iodooctadecane were purchased from Tokyo Chemical Industry Corporation (Tokyo, Japan). L-Ascorbic acid (AA), L-ascorbic acid sodium salt (AANa), acetyl chloride, chloromethyl methyl ether, potassium carbonate, hydrochloric acid (HCl), oxatomide, and p-nitrophenyl-2-acetamido-2-deoxy-β-D-glucopyranoside (PNAG) were purchased from FUJIFILM Wako Pure Chemical Corporation (Osaka, Japan). Diaion HP20 (Mitsubishi Chemical Corporation, Tokyo, Japan), Wakogel C-200 (FUJIFILM Wako Pure Chemical Corporation), Silica Gel 60 (Merck KGaA, Darmstadt, Germany), and TOYOPEARL HW-40C (Tosoh Corporation, Tokyo, Japan) were utilized for column chromatography. Monoclonal anti-dinitrophenyl antibody (DNP-IgE) and dinitrophenyl-labeled human serum albumin (DNP-HSA) were obtained from Sigma-Aldrich Co., St. Louis, MO, USA. All of the chemicals used were of the highest grade commercially available.

### 3.2. Instruments

NMR experiments were performed using a Varian NMR System 600-MHz instrument (Palo Alto, CA, USA) and JEOL NMR Systems 400-MHz and 500-MHz instruments (Tokyo, Japan). The chemical shifts are reported in ppm, and each coupling constant (*J*) is reported in Hz. Electron spray ionization (ESI) high-resolution mass spectra were acquired on a Bruker Daltonics MicrOTOF II instrument (Bremen, Germany) and a Waters LCT Premier instrument (Milford, MA, USA).

### 3.3. Synthesis of 2-O-Butyl-l-ascorbic Acid (***1***)

2-*O*-Alkyl-l-ascorbic acid (2-AlkylAA) was synthesized according to a method from Kato et al. 5,6-Isopropylidene-3-methoxymethyl-AA was synthesized by the method described by Kato et al. [13].

5,6-Isopropylidene-3-methoxymethyl-AA (3.98 g, 15.3 mmol) in THF (14 mL)/dimethyl sulfoxide (DMSO) (15 mL) was added to potassium carbonate (2.34 g, 16.9 mmol) and 1-iodobutane (1.90 mL, 16.7 mmol), and the reaction mixture was stirred at 50 °C. After 2.5 h, the reaction mixture was diluted with H_2_O (50 mL), neutralized with conc. HCl, and extracted with EtOAc (100 mL). The EtOAc layer was washed with H_2_O, dried over anhydrous sodium sulfate, and concentrated in vacuo. The obtained EtOAc fraction was purified using Wakogel C-200 eluted with *n*-hexane/EtOAc (70/30, *v*/*v*) to obtain 5,6-isopropylidene-3-methoxymethyl-2-*O*-butylAA (2.58 g, 8.2 mmol). 5,6-Isopropylidene-3-methoxymethyl-2-*O*-butylAA (2.58 g) was dissolved in an EtOH (12 mL)/1 M HCl aqueous solution (4 mL), and the solution was stirred at 80 °C. After 1 h, the reaction mixture was concentrated and dried in vacuo. The resulting product was purified using Diaion HP20 eluted stepwise with MeOH/H_2_O/formic acid (0/99.5/0.5, 20/79.5/0.5, 40/59.5/0.5, 60/39.5/0.5, 80/19.5/0.5, *v*/*v*). The eluted fractions in MeOH/H_2_O (60/39.5/0.5, 80/19.5/0.5, *v*/*v*) were combined and concentrated (1.60 g), and then recrystallized with IPE/EtOAc to yield compound **1** (1.05 g). The total yield from AA was 10.5%. ^1^H-NMR (500 MHz, CD_3_OD): δ 4.86 (d, *J* = 1.9 Hz, 1H), 4.06–3.98 (m, 2H), 3.95 (dt, *J* = 1.9, 7.0 Hz, 1H), 3.71 (d, *J* = 7.0 Hz, 2H), 1.71 (quin, *J* = 7.2 Hz, 2H), 1.48 (sext, *J* = 7.2 Hz, 2H), 0.99 (t, *J* = 7.2 Hz, 3H); ^13^C-NMR (125 MHz, CD_3_OD): δ 173.2, 161.8, 122.0, 76.9, 72.9, 70.5, 63.4, 32.8, 19.9, 14.2; ESI-HRMS [M − H]^−^ calcd for C_10_H_15_O_6_. 231.0874; found, 231.0868. ^1^H-NMR, ^13^C-NMR, and HRMS spectra data are shown in Supplementary Materials (Figures S1–S3).

### 3.4. Synthesis of 2-O-Octyl-l-ascorbic Acid (***2***)

5,6-Isopropylidene-3-methoxymethyl-AA (4.29 g, 16.5 mmol) in THF (14 mL)/DMSO (15 mL) was added to potassium carbonate (2.52 g, 18.2 mmol) and 1-iodooctane (3.30 mL, 18.2 mmol), and the reaction mixture was stirred at 50 °C. After 2.5 h, the reaction mixture was diluted with H_2_O (50 mL), neutralized with conc. HCl, and extracted with EtOAc (100 mL). The EtOAc layer was washed with H_2_O, dried over anhydrous sodium sulfate, and concentrated in vacuo. The obtained EtOAc fraction was purified using Wakogel C-200 eluted with *n*-hexane/EtOAc (80/20, *v*/*v*) to obtain 5,6-isopropylidene-3-methoxymethyl-2-*O*-octylAA (3.13 g, 8.4 mmol). 5,6-Isopropylidene-3-methoxymethyl-2-*O*-octylAA (3.13 g) was dissolved in an EtOH (12 mL)/1 M HCl aqueous solution (4 mL), and the solution was stirred at 80 °C. After 2 h, the reaction mixture was concentrated and dried in vacuo. The reaction mixture was purified using Wakogel C-200 eluted with EtOAc/formic acid (99.5/0.5, *v*/*v*) to obtain a fraction containing compound **2** (0.99 g). The obtained fraction was recrystallized with IPE/EtOAc to yield compound **2** (0.77 g). The total yield from AA was 6.2%. ^1^H-NMR (500 MHz, CD_3_OD): δ 4.88 (d, *J* = 1.8 Hz, 1H), 4.05–3.98 (m, 2H), 3.95 (dt, *J* = 1.8, 7.0 Hz, 1H), 3.71 (d, *J* = 7.0 Hz, 2H), 1.72 (quin, *J* = 7.0 Hz, 2H), 1.46–1.34 (m, 10H), 0.94 (t, *J* = 7.0 Hz, 3H); ^13^C-NMR (125 MHz, CD_3_OD): δ 173.1, 161.3, 122.1, 76.8, 73.2, 70.5, 63.4, 33.0, 30.7, 30.5, 30.4, 26.8, 23.7, 14.4; ESI-HRMS [M − H]^−^ calcd for C_14_H_23_O_6_. 287.1500; found, 287.1495. ^1^H-NMR, ^13^C-NMR, and HRMS spectra data are shown in Supplementary Materials (Figures S4–S6).

### 3.5. Synthesis of 2-O-Dodecyl-l-ascorbic Acid (***3***)

5,6-Isopropylidene-3-methoxymethyl-AA (5.94 g, 22.9 mmol) in THF (20 mL)/DMSO (20 mL) was added to potassium carbonate (3.58 g, 25.2 mmol) and 1-iodododecane (6.18 mL, 25.2 mmol), and the reaction mixture was stirred at 50 °C. After 2 h, the reaction mixture was diluted with H_2_O (200 mL), neutralized with conc. HCl, and extracted with EtOAc (200 mL). The EtOAc layer was washed with H_2_O, dried over anhydrous sodium sulfate, and concentrated in vacuo. The obtained EtOAc fraction was purified using Wakogel C-200 eluted with *n*-hexane/EtOAc (70/30, *v*/*v*) to obtain 5,6-isopropylidene-3-methoxymethyl-2-*O*-dodecylAA (5.91 g, 13.8 mmol). 5,6-Isopropylidene-3-methoxymethyl-2-*O*-dodecylAA (5.91 g) was dissolved in an EtOH (8 mL)/1 M HCl aqueous solution (2 mL), and the solution was stirred at 80 °C. After 1 h, the reaction mixture was concentrated and dried in vacuo. The reaction mixture was extracted with EtOAc (100 mL) and washed with H_2_O. Then the EtOAc layer was dried over anhydrous sodium sulfate and concentrated in vacuo. The resulting product was recrystallized with EtOAc to yield compound **3** (1.11 g). The total yield from AA was 7.8%. ^1^H-NMR (500 MHz, CD_3_OD): δ 4.87 (d, *J* = 1.8 Hz, 1H), 4.03–3.99 (m, 2H), 3.95 (dt, *J* = 1.8, 7.0 Hz, 1H), 3.71 (d, *J* = 7.0 Hz, 2H), 1.72 (quin, *J* = 7.0 Hz, 2H), 1.44–1.33 (m, 18H), 0.94 (t, *J* = 7.0 Hz, 3H); ^13^C-NMR (125 MHz, CD_3_OD): δ 173.1, 161.5, 122.0, 76.8, 73.2, 70.5, 63.4, 33.1, 30.8 (3C), 30.7 (2C), 30.6, 30.5, 26.8, 23.7, 14.4; ESI-HRMS [M − H]^−^ calcd for C_18_H_31_O_6_. 343.2126; found, 343.2136. ^1^H-NMR, ^13^C-NMR, and HRMS spectra data are shown in Supplementary Materials (Figures S7–S9).

### 3.6. Synthesis of 2-O-Hexadecyl-l-ascorbic Acid (***4***)

5,6-Isopropylidene-3-methoxymethyl-AA (1.41 g, 5.4 mmol) in THF (10 mL)/DMSO (10 mL) was added to potassium carbonate (0.85 g, 6.0 mmol) and 1-iodohexadecane (1.61 mL, 6.0 mmol), and the reaction mixture was stirred at 50 °C. After 3 h, the reaction mixture was diluted with H_2_O (100 mL), neutralized with conc. HCl, and extracted with EtOAc (100 mL). The EtOAc layer was washed with H_2_O, dried over anhydrous sodium sulfate, and concentrated in vacuo. The obtained EtOAc fraction was purified using Wakogel C-200 eluted with *n*-hexane/EtOAc (70/30, *v*/*v*) to obtain 5,6-isopropylidene-3-methoxymethyl-2-*O*-hexadecylAA (1.84 g, 3.8 mmol). 5,6-Isopropylidene-3-methoxymethyl-2-*O*-hexadecylAA (1.84 g) was dissolved in an EtOH (8 mL)/1 M HCl aqueous solution (2 mL), and the solution was stirred at 80 °C. After 1 h, the reaction mixture was concentrated and dried in vacuo. The reaction mixture was extracted with EtOAc (100 mL) and washed with H_2_O. Then the EtOAc layer was dried over anhydrous sodium sulfate and concentrated in vacuo. The resulting product was recrystallized with EtOAc to yield compound **4** (477.1 mg). The total yield from AA was 12.2%. ^1^H-NMR (600 MHz, CD_3_OD): δ 4.86 (d, *J* = 1.8 Hz, 1H), 4.04–3.98 (m, 2H), 3.95 (dt, *J* = 1.8, 7.2 Hz, 1H), 3.71 (d, *J* = 7.2 Hz, 2H), 1.72 (quin, *J* = 7.2 Hz, 2H), 1.47–1.29 (m, 26H), 0.94 (t, *J* = 6.6 Hz, 3H); ^13^C-NMR (151 MHz, CD_3_OD): δ 173.2, 161.7, 122.0, 76.9, 73.2, 70.5, 63.4, 33.1, 30.8 (7C), 30.7 (3C), 30.6, 26.8, 23.7, 14.4; ESI-HRMS [M + H]^+^ calcd for C_22_H_41_O_6_. 401.2903; found, 401.2918. ^1^H-NMR, ^13^C-NMR, and HRMS spectra data are shown in Supplementary Materials (Figures S10–S12).

### 3.7. Synthesis of 2-O-Octadecyl-l-ascorbic Acid (***5***)

5,6-Isopropylidene-3-methoxymethyl-AA (3.95 g, 15.2 mmol) in THF (10 mL)/DMSO (12 mL) was added to potassium carbonate (1.90 g, 13.8 mmol) and 1-iodooctadecane (5.23 g, 13.8 mmol), and the reaction mixture was stirred at 50 °C. After 2.5 h, the reaction mixture was diluted with H_2_O (40 mL), neutralized with conc. HCl, and extracted with *n*-hexane (80 mL). The *n*-hexane layer was washed with H_2_O, dried over anhydrous sodium sulfate, and concentrated in vacuo. The obtained *n*-hexane fraction was purified using Wakogel C-200 eluted with *n*-hexane/EtOAc (80/20, *v*/*v*) to obtain 5,6-isopropylidene-3-methoxymethyl-2-*O*-octadecylAA (3.13 g, 6.1 mmol). 5,6-Isopropylidene-3-methoxymethyl-2-*O*-octadecylAA (2.52 g, 4.9 mmol) was dissolved in an EtOH (9 mL)/1 M HCl aqueous solution (3 mL), and the solution was stirred at 80 °C. After 1 h, the reaction mixture was concentrated and dried in vacuo. The resulting product was recrystallized with EtOH to yield compound **5** (1.55 g). The total yield from AA is 10.4%. ^1^H-NMR (600 MHz, DMSO-*d*_6_): δ 4.73 (brs, 1H), 3.87–3.81 (m, 2H), 3.75 (t, *J* = 6.4 Hz, 1H), 3.45–3.37 (m, 2H), 1.55 (quin, *J* = 6.8 Hz, 2H), 1.37–1.17 (m, 30H), 0.84 (t, *J* = 6.4 Hz, 3H); ^13^C-NMR (151 MHz, DMSO-*d*_6_): δ 170.1, 159.7, 119.9, 74.8, 71.2, 68.5, 61.9, 31.5, 29.2 (11C), 29.0, 28.9, 25.4, 22.3, 14.1; ESI-HRMS [M − H]^−^ calcd for C_24_H_43_O_6_. 427.3065; found, 427.3052. ^1^H-NMR, ^13^C-NMR, and HRMS spectra data are shown in Supplementary Materials (Figures S13–S15).

### 3.8. Synthesis of 3-O-Butyl-l-ascorbic Acid (***6***)

3-*O*-Alkyl-l-ascorbic acid (3-AlkylAA) was synthesized in our laboratory using a single-step method described previously [18]. L-Ascorbic acid sodium salt (AANa) (1.19 g, 6.0 mmol) in DMSO (30 mL) was added to 1-iodobutane (900 μL, 8.4 mmol), and the reaction mixture was stirred at 50 °C. After 3 h, the reaction mixture was diluted with H_2_O (30 mL). The obtained reaction mixture was purified using Diaion HP20 eluted stepwise with MeOH/H_2_O (0/100, 20/80, 40/60, 80/20, *v*/*v*). The eluted fractions in MeOH/H_2_O (80/20, *v*/*v*) were combined and concentrated in vacuo (1.18 g). The concentrated fraction (1.18 g) was purified using Wakogel C-200 eluted with toluene/acetone (55/45, *v*/*v*) to obtain the fraction containing compound **6** (1.01 g). Moreover, the resulting fraction was purified using TOYOPEARL HW-40C eluted with MeOH/H_2_O (25/75, *v*/*v*) to isolate compound **6** (714.1 mg, 51.3%). ^1^H-NMR (400 MHz, CD_3_OD): δ 4.76 (d, *J* = 1.8 Hz, 1H), 4.55–4.44 (m, 2H), 3.83 (dt, *J* = 1.8, 7.4 Hz, 1H), 3.64 (d, *J* = 7.4 Hz, 2H), 1.72 (quin, *J* = 7.0 Hz, 2H), 1.45 (sext, *J* = 7.0 Hz, 2H), 0.96 (t, *J* = 7.0 Hz, 3H); ^13^C-NMR (100 MHz, CD_3_OD): δ 173.2, 152.2, 120.4, 76.6, 72.4, 70.5, 63.4, 32.9, 19.8, 14.1; ESI-HRMS [M − H]^−^ calcd for C_10_H_15_O_6_. 231.0869; found, 231.0847. ^1^H-NMR, ^13^C-NMR, and HRMS spectra data are shown in Supplementary Materials (Figures S16–S18).

### 3.9. Synthesis of 3-O-Octyl-l-ascorbic Acid (***7***)

AANa (1.19 g, 6.0 mmol) in DMSO (30 mL) was added to 1-iodooctane (1.12 mL, 7.2 mmol), and the reaction mixture was stirred at 50 °C. After 12 h, the reaction mixture was diluted with H_2_O (270 mL) and extracted with EtOAc (300 mL). The EtOAc layer was washed with H_2_O twice, dried over anhydrous sodium sulfate, and concentrated in vacuo. The obtained EtOAc fraction was purified using Wakogel C-200 eluted with toluene/acetone/MeOH (70/25/5, *v*/*v*) to obtain the fraction containing compound **7**. Then, the fraction was also purified using Silica gel 60 eluted with toluene/acetone (70/30, *v*/*v*) to obtain compound **7** (702.1 mg, 40.6%). ^1^H-NMR (500 MHz, CD_3_OD): δ 4.76 (d, *J* = 2.0 Hz, 1H), 4.54–4.43 (m, 2H), 3.83 (brt, *J* = 7.0 Hz, 1H), 3.64 (d, *J* = 7.0 Hz, 2H), 1.74 (quin, *J* = 7.0 Hz, 2H), 1.43–1.25 (m, 10H), 0.90 (t, *J* = 7.0 Hz, 3H); ^13^C-NMR (125 MHz, CD_3_OD): δ 173.3, 152.2, 120.5, 76.6, 72.2, 70.5, 63.5, 33.0, 30.9, 30.4, 30.3, 26.7, 23.7, 14.4; ESI-HRMS [M − H]^−^ calcd for C_14_H_23_O_6_. 287.1495; found, 287.1494. ^1^H-NMR, ^13^C-NMR, and HRMS spectra data are shown in Supplementary Materials (Figures S19–S21).

### 3.10. Synthesis of 3-O-Dodecyl-l-ascorbic Acid (***8***)

AANa (1.19 g, 6.0 mmol) in DMSO (30 mL) was added to 1-iodododecane (1.8 mL, 7.2 mmol), and the reaction mixture was stirred at 50 °C. After 3 h, the reaction mixture was diluted with H_2_O (60 mL) and extracted with EtOAc (90 mL). The EtOAc layer was washed with H_2_O, dried over anhydrous sodium sulfate, and concentrated in vacuo. The obtained EtOAc fraction was purified using Wakogel C-200 eluted with toluene/MeOH (90/10, *v*/*v*) to obtain the fraction containing compound **8**. The resulting product was recrystallized with IPE to yield compound **8** (0.83 g, 40.0%). ^1^H-NMR (600 MHz, CD_3_OD): δ 4.81 (d, *J* = 1.8 Hz, 1H), 4.58–4.49 (m, 2H), 3.88 (dt, *J* = 1.8, 7.2 Hz, 1H), 3.69 (d, *J* = 7.2 Hz, 2H), 1.79 (quin, *J* = 7.2 Hz, 2H), 1.49–1.29 (m, 18H), 0.94 (t, *J* = 7.2 Hz, 3H); ^13^C-NMR (151 MHz, CD_3_OD): δ 173.3, 152.3, 120.5, 76.7, 72.7, 70.6, 63.5, 33.1, 30.9, 30.7 (4C), 30.5 (2C), 26.7, 23.8, 14.4; ESI-HRMS [M − H]^−^ calcd for C_18_H_31_O_6_. 343.2126; found, 343.2123. ^1^H-NMR, ^13^C-NMR, and HRMS spectra data are shown in Supplementary Materials (Figures S22–S24).

### 3.11. Synthesis of 3-O-Hexadecyl-l-ascorbic Acid (***9***)

AANa (1.19 g, 6.0 mmol) in DMSO (30 mL) was added to 1-iodohexadecane (2.6 mL, 8.4 mmol), and the reaction mixture was stirred at 50 °C. After 3 h, the reaction mixture was diluted with H_2_O (300 mL) and extracted with EtOAc (300 mL). The EtOAc layer was washed with H_2_O, dried over anhydrous sodium sulfate, and concentrated in vacuo. The obtained EtOAc fraction was purified using Wakogel C-200 eluted with toluene/acetone (55/45, *v*/*v*) to obtain the fraction containing compound **9**. The resulting product was recrystallized with EtOAc to yield compound **9** (323.8 mg, 13.5%). ^1^H-NMR (600 MHz, CD_3_OD): δ 4.75 (d, *J* = 1.2 Hz, 1H), 4.53–4.43 (m, 2H), 3.82 (dt, *J* = 1.2, 6.6 Hz, 1H), 3.63 (d, *J* = 6.6 Hz, 2H), 1.73 (quin, *J* = 7.8 Hz, 2H), 1.43–1.23 (m, 26H), 0.88 (t, *J* = 7.2 Hz, 3H); ^13^C-NMR (151 MHz, CD_3_OD): δ 171.8, 150.8, 119.0, 75.2, 71.3, 69.2, 62.0, 31.6, 29.5, 29.4 (5C), 29.3 (3C), 29.0 (2C), 25.2, 22.3, 13.0; ESI-HRMS [M − H]^−^ calcd for C_22_H_39_O_6_. 399.2752; found, 399.2765. ^1^H-NMR, ^13^C-NMR, and HRMS spectra data are shown in Supplementary Materials (Figures S25–S27).

### 3.12. Synthesis of 3-O-Octadecyl-l-ascorbic Acid (***10***)

AANa (1.19 g, 6.0 mmol) in DMSO (30 mL) was added to 1-iodooctadecane (3.25 g, 8.4 mmol), and the reaction mixture was stirred at 50 °C. After 3 h, the reaction mixture was diluted with H_2_O (100 mL) and extracted with EtOAc (100 mL). The EtOAc layer was washed with H_2_O, dried over anhydrous sodium sulfate, and concentrated in vacuo. The obtained EtOAc fraction was purified by Wakogel C-200 eluted with toluene/acetone (55/45, *v*/*v*) to obtain the fraction containing compound **10**. The resulting product was recrystallized with IPE to yield compound **10** (721.9 mg, 28.2%). ^1^H-NMR (600 MHz, CD_3_OD): δ 4.80 (d, *J* = 1.8 Hz, 1H), 4.58–4.49 (m, 2H), 3.88 (dt, *J* = 1.8, 6.6 Hz, 1H), 3.70 (d, *J* = 6.6 Hz, 2H), 1.79 (quin, *J* = 6.6 Hz, 2H), 1.50–1.29 (m, 46H), 0.94 (t, *J* = 7.2 Hz, 3H); ^13^C-NMR (151 MHz, CD_3_OD): δ 173.2, 152.3, 120.5, 76.7, 72.8, 70.7, 63.5, 33.1, 30.8 (10C), 30.7 (2C), 30.4, 26.7, 23.7, 14.4; ESI-HRMS [M − H]^−^ calcd for C_24_H_43_O_6_. 427.3065; found, 427.3086. ^1^H-NMR, ^13^C-NMR, and HRMS spectra data are shown in Supplementary Materials (Figures S28–S30).

### 3.13. Synthesis of 6-Deoxy-6-amino-3-O-dodecyl-l-ascorbic Acid (***11***)

6-Substituted 3-*O*-DodecylAA was synthesized according to a method from Andrews et al. [24]. Compound **8** (5.50 g, 16.0 mmol) in acetic acid (10 mL) was added to HBr (6.3 mL, 32.0 mmol), and the reaction mixture was stirred at 30 °C. After 15 h, the reaction mixture was concentrated and added to EtOH/2N HCl solution (70/30, *v*/*v*) and the reaction mixture was stirred at 60 °C. After 7 h, the reaction product was concentrated, diluted with H_2_O (50 mL), and extracted with EtOAc (100 mL). The EtOAc layer was washed with H_2_O, dried over anhydrous sodium sulfate, and concentrated in vacuo. The obtained EtOAc fraction was purified using Wakogel C-200 eluted with toluene/acetone (90/10, *v*/*v*) to obtain the fraction containing 6-deoxy-6-bromo-3-*O*-dodecyl-l-ascorbic acid. The resulting product was recrystallized with *n*-hexane to yield 6-deoxy-6-bromo-3-*O*-dodecyl-l-ascorbic acid. (3.34 g, 51.3%).

6-Deoxy-6-bromo-3-*O*-dodecyl-l-ascorbic acid (600.0 mg, 1.47 mmol) in EtOH (12 mL) was added to sodium azide (191.1 mg, 2.94 mmol) and sodium carbonate (623.3 mg, 5.88 mmol), and the reaction mixture was stirred at 30 °C. After 20 h, the reaction product was concentrated, diluted with H_2_O (50 mL), and extracted with EtOAc (50 mL × 1, 100 mL × 2). The EtOAc layer was washed with H_2_O, dried over anhydrous sodium sulfate, and concentrated in vacuo. The obtained EtOAc fraction was purified using Wakogel C-200 eluted with toluene/acetone (90/10, *v*/*v*) to isolate 6-deoxy-6-azide-3-*O*-dodecyl-l-ascorbic acid (230.3 mg, 40.1%).

6-Deoxy-6-azide-3-*O*-dodecyl-l-ascorbic acid (317.0 mg, 0.82 mmol) in EtOH (3 mL) was added to Pd-C (39.7 mg) for a catalyst. The reaction mixture was stirred vigorously at room temperature under an H_2_ atmosphere for 8 h. After the reaction, the catalyst was removed using filtration, and the filtrate was concentrated and dried in vacuo. The obtained product was purified using Wakogel C-200 eluted with EtOAc/MeOH/formic acid (80/19.9/0.1, *v*/*v*/*v*) to isolate compound **11** (73.2 mg, 25.6%). The total yield from AANa was 3.3%. ^1^H-NMR (500 MHz, CD_3_OD): δ 4.67 (d, *J* = 2.0 Hz, 1H), 4.57–4.43 (m, 2H), 4.03–4.0 (m, 1H), 3.15 (dd, *J* = 4.0, 13.0 Hz, 1H), 3.04 (dd, *J* = 9.0, 13.0 Hz, 1H), 1.74 (quin, *J* = 6.5 Hz, 2H), 1.28 (m, 18H), 0.89 (t, *J* = 6.5 Hz, 3H); ^13^C-NMR (125 MHz, CD_3_OD); δ 172.6, 150.9, 121.0, 77.9, 72.9, 67.5, 43.6, 33.1, 30.9, 30.8 (2C), 30.7 (2C), 30.5 (2C), 26.7, 23.7, 14.4; ESI-HRMS [M + H]^+^ calcd for C_18_H_34_NO_5_. 344.2437; found, 344.2437. ^1^H-NMR, ^13^C-NMR, and HRMS spectra data are shown in Supplementary Materials (Figures S31–S33).

### 3.14. Synthesis of 3-Deoxy-3-dodecylamino-l-ascorbic Acid (***12***)

Compound **12** was synthesized according to a method of Pischetsrieder et al. [25]. AA (1.50 g, 8.52 mmol) in *N*,*N*-dimethylformamide (DMF) (30 mL) was added to dodecylamine (3.16 g, 17.04 mmol), and the reaction mixture was stirred at 100 °C. After 2 h, the reaction product was diluted with H_2_O (300 mL) and extracted with EtOAc (300 mL). The EtOAc layer was washed with H_2_O, dried over anhydrous sodium sulfate, and concentrated in vacuo. The EtOAc layer was purified using Wakogel C-200 eluted with EtOAc to obtain the fraction containing compound **12**. The fraction was also purified using Wakogel C-200 eluted with toluene/MeOH (90/10, *v*/*v*) to isolate compound **12** (72.8 mg, 2.5%). ^1^H-NMR (600 MHz, CD_3_OD); δ 4.73 (d, *J* = 1.8 Hz, 1H), 3.88 (dt, *J* = 1.8, 6.6 Hz, 1H), 3.66–3.60 (m, 2H), 3.51–3.39 (m, 2H), 1.60 (quin, *J* = 7.2 Hz, 2H), 1.39–1.24 (m, 18H), 0.90 (t, *J* = 6.9 Hz, 3H); ^13^C-NMR (151 MHz, CD_3_OD); δ 173.0, 147.4, 113.1, 75.0, 70.5, 62.0, 43.2, 31.7, 29.5, 29.3 (4C), 29.1 (2C), 26.4, 22.3, 13.0; ESI-HRMS [M − H]^−^ calcd for C_18_H_32_NO_5_. 342.2286; found, 342.2282. ^1^H-NMR, ^13^C-NMR, and HRMS spectra data are shown in Supplementary Materials (Figures S34–S36).

### 3.15. Antigen-Stimulated Degranulation Assay

The inhibitory activities of synthesized AA derivatives against antigen-stimulated degranulation from RBL-2H3 cells were investigated by modifying the method by Watanabe et al. [26]. Briefly, RBL-2H3 cells were plated at 5.0 × 10^4^ cells/200 μL/well in 96-well plates and incubated in a humidified atmosphere of 5% CO_2_ at 37 °C. After 24 h, the cells were incubated in 100 μL of DMEM containing 50 ng/mL of monoclonal anti-DNP IgE for 2 h. The incubated cells were washed with modified Tyrode (MT) buffer (pH 7.3) before 90 μL of each of the AA derivatives or oxatomide was added. The AA derivatives and oxatomide were dissolved in DMSO and diluted with MT buffer (final DMSO concentration: 0.25%). After 20 min of incubation, 10 μL of DNP-HSA (final concentration: 50 ng/mL) was added to the cells, and the cultures were incubated for 1 h. After the supernatants were collected, the cells were lysed with 100 μL of MT buffer containing 0.1% Triton X-100. The β-hexosaminidase activities of the supernatants and cell lysates were measured using the method reported by Demo et al. [27]. Each 20 μL of an aliquot of the supernatant or cell lysate was mixed with a 40-μL volume of 3.3 mM PNAG in 100 mM citrate buffer (pH 4.5), and the mixture was incubated at 37 °C for 90 min. Each reaction was terminated with the addition of 40 μL of 2 M glycine buffer (pH 10.4), and the absorbance of each well at 405 nm was measured using a microplate reader (Varioskan FC from Thermo Fisher Scientific, Waltham, MA, USA).

Degranulation ratio (%) = [St − Sb/{(St − Sb) + C}] × 100.

St, Sb, and C in this equation express the absorbance of sample-treated (St), sample-blank (Sb), and cell lysate (C) with the stimulant only, respectively.

### 3.16. Calcium Ionophore-Stimulated Degranulation Assay

The inhibitory activities of compounds **4**, **5**, **8** and **9** and oxatomide against calcium ionophore-stimulated degranulation from RBL-2H3 cells were investigated according to a previously published method [28]. Briefly, RBL-2H3 cells were cultured at 5.0 × 10^4^ cells/200 μL/well in a 96-well plate in a humidified atmosphere of 5% CO_2_ at 37 °C for 24 h. The cells were washed with MT buffer before the addition of 90 μL of the AA derivatives or oxatomide as described in the previous section. After 20 min of incubation, 10 μL of the calcium ionophore A23187 (final concentration: 1 μM) was added, and the cultures were incubated for 1 h. After the supernatants were collected, and the cells were lysed with MT buffer containing 0.1% Triton X-100. The degranulation assay was performed as described in the previous section.

### 3.17. PCA Reaction in Mice

The inhibitory activity against the PCA reaction was evaluated with a slightly modified method of our previous report [12]. The IgE-stimulated PCA reaction was performed as follows. Each mouse (ICR mouse, 7 Ws, male) was injected with 20 μL of anti-DNP-IgE antibody (5 mg/mL) in the ears. After 24 h, oxatomide (100 μmol/kg, b.w.) and compound **8** (100 μmol/kg or 200 μmol/kg, b.w.) were administered orally. The control group was administered 5% EtOH/D-PBS. After 2 h of oral administration, saline containing DNP-HSA and Evan’s blue was injected intravenously in the tail of the mice. After 30 min, each ear was removed and dissolved in 1 M KOH solution. The extravasated Evan’s blue dye was extracted with an acetone/0.4 M phosphoric acid (13/5, *v*/*v*) solution and centrifuged. The absorbance at 620 nm was measured to calculate the percentage of inhibitory activity against the PCA reaction. The experiments were approved by the Committee for Ethics in Animal Experiments of the Prefectural University of Hiroshima (16SA-019).

## 4. Conclusions

To find simple monosubstituted AA derivatives with antiallergic activity, 2-*O*-AlkylAA or 3-*O*-AlkylAA derivatives with alkyl groups of various lengths introduced at the C-2 or C-3 positions of AA were synthesized, and their inhibitory activities against antigen-stimulated degranulation were evaluated. Among the evaluated 2-*O*-AlkylAA and 3-*O*-AlkylAA derivatives, compound **8** with a dodecyl group introduced at the C-3 position of AA showed the strongest activity. Compound **8** also inhibited calcium ionophore-stimulated degranulation. Furthermore, based on compound **8**, compound **11** with the hydroxyl group at the C-6 position of compound **8** substituted with an amino group and compound **12** with the dodecyl group at the C-3 position of compound **8** substituted with a dodecylamino group were synthesized, and their antigen-stimulated degranulation inhibitory activities were investigated. The investigation revealed that compound **8** had stronger inhibitory activity than the inhibitory activities of compounds **11** and **12**. Orally administered compound **8** also strongly inhibited the PCA reaction in mice with potency comparable to that of oxatomide, which is used as an antiallergic agent. Compound **8**, a simple monosubstituted AA derivative, is expected to be developed as an antiallergic agent.

## Data Availability

Data are contained within the article and Supplementary Materials.

## Supplementary Materials

The following supporting information can be downloaded at https://www.mdpi.com/article/10.3390/molecules29010069/s1, Figure S1: ^1^H-NMR spectrum of 2-*O*-butyl-l-ascorbic acid (**1**); Figure S2: ^13^C-NMR spectrum of 2-*O*-butyl-l-ascorbic acid (**1**); Figure S3: HRMS spectrum of 2-*O*-butyl-l-ascorbic acid (**1**); Figure S4: ^1^H-NMR spectrum of 2-*O*-octyl-l-ascorbic acid (**2**); Figure S5: ^13^C-NMR spectrum of 2-*O*-octyl-l-ascorbic acid (**2**); Figure S6: HRMS spectrum of 2-*O*-octyl-l-ascorbic acid (**2**); Figure S7: ^1^H-NMR spectrum of 2-*O*-dodecyl-l-ascorbic acid (**3**); Figure S8: ^13^C-NMR spectrum of 2-*O*-dodecyl-l-ascorbic acid (**3**); Figure S9: HRMS spectrum of 2-*O*-dodecyl-l-ascorbic acid (**3**); Figure S10: ^1^H-NMR spectrum of 2-*O*-hexadecyl-l-ascorbic acid (**4**); Figure S11: ^13^C-NMR spectrum of 2-*O*-hexadecyl-l-ascorbic acid (**4**); Figure S12: HRMS spectrum of 2-*O*-hexadecyl-l-ascorbic acid (**4**); Figure S13: ^1^H-NMR spectrum of 2-*O*-octadecyl-l-ascorbic acid (**5**); Figure S14: ^13^C-NMR spectrum of 2-*O*-octadecyl-l-ascorbic acid (**5**); Figure S15: HRMS spectrum of 2-*O*-octadecyl-l-ascorbic acid (**5**); Figure S16: ^1^H-NMR spectrum of 3-*O*-butyl-l-ascorbic acid (**6**); Figure S17: ^13^C-NMR spectrum of 3-*O*-butyl-l-ascorbic acid (**6**); Figure S18: HRMS spectrum of 3-*O*-butyl-l-ascorbic acid (**6**); Figure S19: ^1^H-NMR spectrum of 3-*O*-octyl-l-ascorbic acid (**7**); Figure S20: ^13^C-NMR spectrum of 3-*O*-octyl-l-ascorbic acid (**7**); Figure S21: HRMS spectrum of 3-*O*-octyl-l-ascorbic acid (**7**); Figure S22: ^1^H-NMR spectrum of 3-*O*-dodecyl-l-ascorbic acid (**8**); Figure S23: ^13^C-NMR spectrum of 3-*O*-dodecyl-l-ascorbic acid (**8**); Figure S24: HRMS spectrum of 3-*O*-dodecyl-l-ascorbic acid (**8**); Figure S25: ^1^H-NMR spectrum of 3-*O*-hexadecyl-l-ascorbic acid (**9**); Figure S26: ^13^C-NMR spectrum of 3-*O*-hexadecyl-l-ascorbic acid (**9**); Figure S27: HRMS spectrum of 3-*O*-hexadecyl-l-ascorbic acid (**9**); Figure S28: ^1^H-NMR spectrum of 3-*O*-octadecyl-l-ascorbic acid (**10**); Figure S29: ^13^C-NMR spectrum of 3-*O*-octadecyl-l-ascorbic acid (**10**); Figure S30: HRMS spectrum of 3-*O*-octadecyl-l-ascorbic acid (**10**); Figure S31: ^1^H-NMR spectrum of 6-deoxy-6-amino-3-*O*-dodecyl-l-ascorbic acid (**11**); Figure S32: ^13^C-NMR spectrum of 6-deoxy-6-amino-3-*O*-dodecyl-l-ascorbic acid (**11**); Figure S33: HRMS spectrum of 6-deoxy-6-amino-3-*O*-dodecyl-l-ascorbic acid (**11**); Figure S34: ^1^H-NMR spectrum of 3-deoxy-3-dodecylamino-l-ascorbic acid (**12**); Figure S35: ^13^C-NMR spectrum of 3-deoxy-3-dodecylamino-l-ascorbic acid (**12**); Figure S36: HRMS spectrum of 3-deoxy-3-dodecylamino-l-ascorbic acid (**12**).
