# Supplementary material for: Antiallergic Activity of 3-O-Dodecyl-l-ascorbic Acid
Source: Molecules. 2023 Dec 21;29(1):69. doi: 10.3390/molecules29010069 (PMC10779884; doi:10.3390/molecules29010069)
Supplement: Supplementary file 1 [file molecules-29-00069-s001.zip › molecules-2768460-supplementary.docx]

Supplementary Materials

**Antiallergic Activity of 3-*O*-Dodecyl-L-ascorbic Acid**

Takeru Koga ^1^, Naoaki Kawahara ^2^, Mei Aburada ^3^, Asako Ono ^3^, Shiori Mae ^3^, Aina Yoshida ^2^, Yuji Iwaoka ^4^, Hideyuki Ito ^4^, Akihiro Tai ^1,3,^*

^1^ *Graduate School of Technology, Industrial and Social Sciences, Tokushima University,* 2-1 Minamijosan-jima-cho, Tokushima 770-8513, Japan

^2^ *Graduate School of Sciences and Technology for Innovation, Tokushima University,* 2-1 Minamijosanjima-cho, Tokushima 770-8513, Japan

^3^ *Faculty of Life and Environmental Sciences, Prefectural University of Hiroshima,* 5562 Nanatsuka-cho, Shobara, Hiroshima 727-0023, Japan

^4^ *Faculty of Health and Welfare Science, Okayama Prefectural University, 111 Kuboki,* Soja, Okayama 719-1197, Japan

*** Corresponding author**

*E-mail address*: [atai@tokushima-u.ac.jp](mailto:atai@tokushima-u.ac.jp)

# Contents:

**Figure S1.** ^1^H-NMR spectrum of 2-*O*-butyl-L-ascorbic acid (**1**) **Figure S2.** ^13^C-NMR spectrum of 2-*O*-butyl-L-ascorbic acid (**1**) **Figure S3.** HRMS spectrum of 2-*O*-butyl-L-ascorbic acid (**1**)

**Figure S4.** ^1^H-NMR spectrum of 2-*O*-octyl-L-ascorbic acid (**2**) **Figure S5.** ^13^C-NMR spectrum of 2-*O*-octyl-L-ascorbic acid (**2**) **Figure S6.** HRMS spectrum of 2-*O*-octyl-L-ascorbic acid (**2**) **Figure S7.** ^1^H-NMR spectrum of 2-*O*-dodecyl-L-ascorbic acid (**3**) **Figure S8.** ^13^C-NMR spectrum of 2-*O*-dodecyl-L-ascorbic acid (**3**) **Figure S9.** HRMS spectrum of 2-*O*-dodecyl-L-ascorbic acid (**3**)

**Figure S10.** ^1^H-NMR spectrum of 2-*O*-hexadecyl-L-ascorbic acid (**4**) **Figure S11.** ^13^C-NMR spectrum of 2-*O*-hexadecyl-L-ascorbic acid (**4**) **Figure S12.** HRMS spectrum of 2-*O*-hexadecyl-L-ascorbic acid (**4**) **Figure S13.** ^1^H-NMR spectrum of 2-*O*-octadecyl-L-ascorbic acid (**5**) **Figure S14.** ^13^C-NMR spectrum of 2-*O*-octadecyl-L-ascorbic acid (**5**) **Figure S15.** HRMS spectrum of 2-*O*-octadecyl-L-ascorbic acid (**5**) **Figure S16.** ^1^H-NMR spectrum of 3-*O*-butyl-L-ascorbic acid (**6**) **Figure S17.** ^13^C-NMR spectrum of 3-*O*-butyl-L-ascorbic acid (**6**) **Figure S18.** HRMS spectrum of 3-*O*-butyl-L-ascorbic acid (**6**) **Figure S19.** ^1^H-NMR spectrum of 3-*O*-octyl-L-ascorbic acid (**7**) **Figure S20.** ^13^C-NMR spectrum of 3-*O*-octyl-L-ascorbic acid (**7**) **Figure S21.** HRMS spectrum of 3-*O*-octyl-L-ascorbic acid (**7**)

**Figure S22.** ^1^H-NMR spectrum of 3-*O*-dodecyl-L-ascorbic acid (**8**) **Figure S23.** ^13^C-NMR spectrum of 3-*O*-dodecyl-L-ascorbic acid (**8**) **Figure S24.** HRMS spectrum of 3-*O*-dodecyl-L-ascorbic acid (**8**) **Figure S25.** ^1^H-NMR spectrum of 3-*O*-hexadecyl-L-ascorbic acid (**9**) **Figure S26.** ^13^C-NMR spectrum of 3-*O*-hexadecyl-L-ascorbic acid (**9**) **Figure S27.** HRMS spectrum of 3-*O*-hexadecyl-L-ascorbic acid (**9**)

**Figure S28.** ^1^H-NMR spectrum of 3-*O*-octadecyl-L-ascorbic acid (**10**) **Figure S29.** ^13^C-NMR spectrum of 3-*O*-octadecyl-L-ascorbic acid (**10**) **Figure S30.** HRMS spectrum of 3-*O*-octadecyl-L-ascorbic acid (**10**)

**Figure S31.** ^1^H-NMR spectrum of 6-deoxy-6-amino-3-*O*-dodecyl-L-ascorbic acid (**11**) **Figure S32.** ^13^C-NMR spectrum of 6-deoxy-6-amino-3-*O*-dodecyl-L-ascorbic acid (**11**) **Figure S33.** HRMS spectrum of 6-deoxy-6-amino-3-*O*-dodecyl-L-ascorbic acid (**11**) **Figure S34.** ^1^H-NMR spectrum of 3-deoxy-3-dodecylamino-L-ascorbic acid (**12**) **Figure S35.** ^13^C-NMR spectrum of 3-deoxy-3-dodecylamino-L-ascorbic acid (**12**) **Figure S36.** HRMS spectrum of 3-deoxy-3-dodecylamino-L-ascorbic acid (**12**)


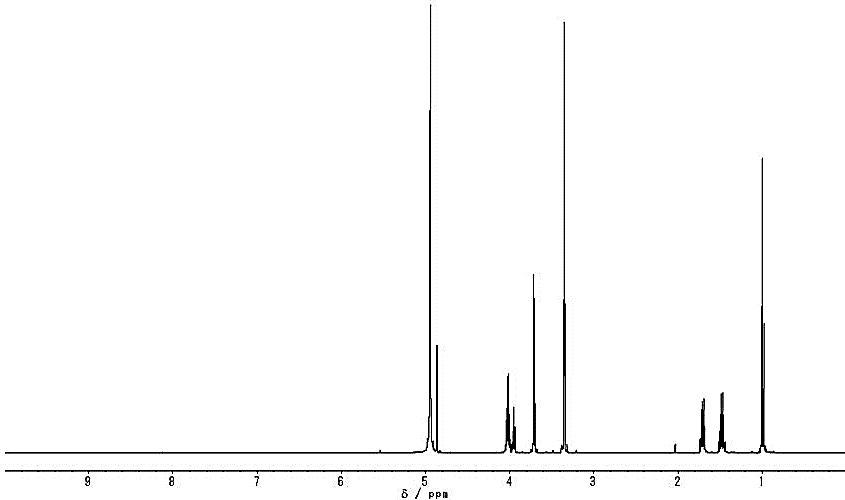


**Figure S1.** ^1^H-NMR spectrum of 2-*O*-butyl-L-ascorbic acid (**1**)


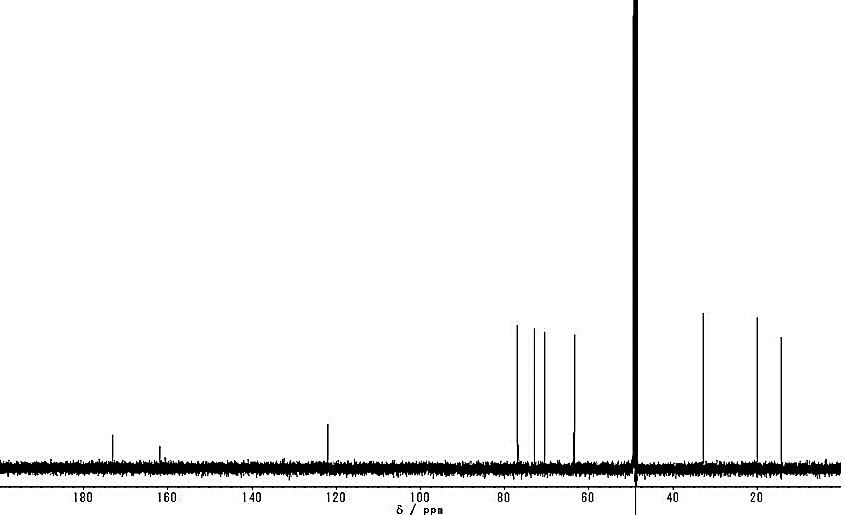


**Figure S2.** ^13^C-NMR spectrum of 2-*O*-butyl-L-ascorbic acid (**1**)


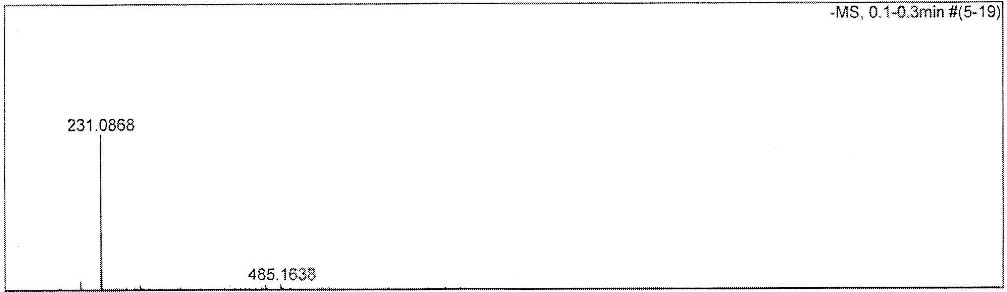


**Figure S3.** HRMS spectrum of 2-*O*-butyl-L-ascorbic acid (**1**)


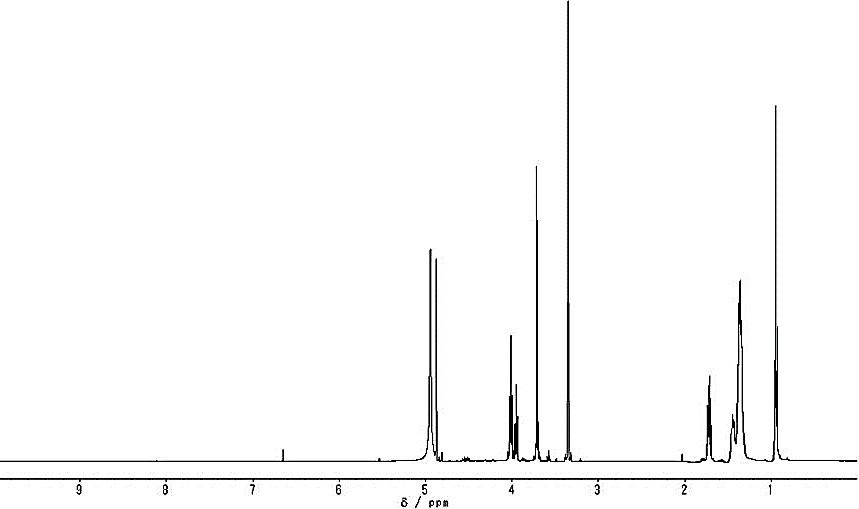


**Figure S4.** ^1^H-NMR spectrum of 2-*O*-octyl-L-ascorbic acid (**2**)


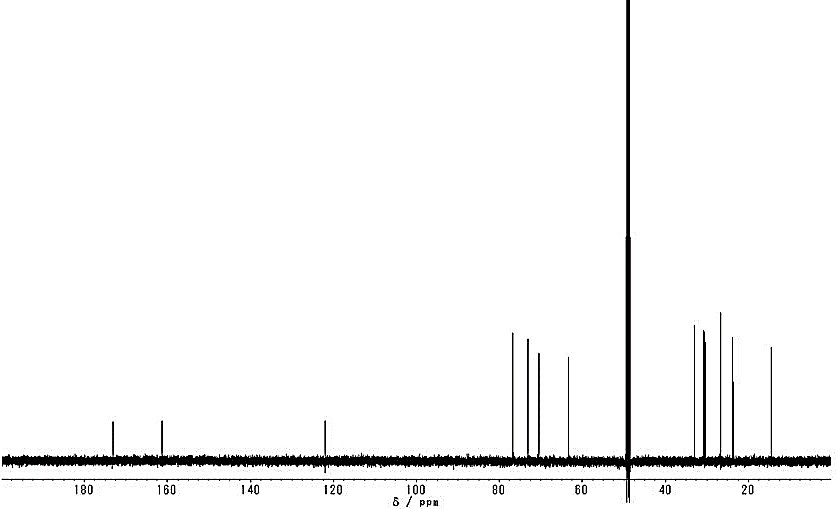


**Figure S5.** ^13^C-NMR spectrum of 2-*O*-octyl-L-ascorbic acid (**2**)


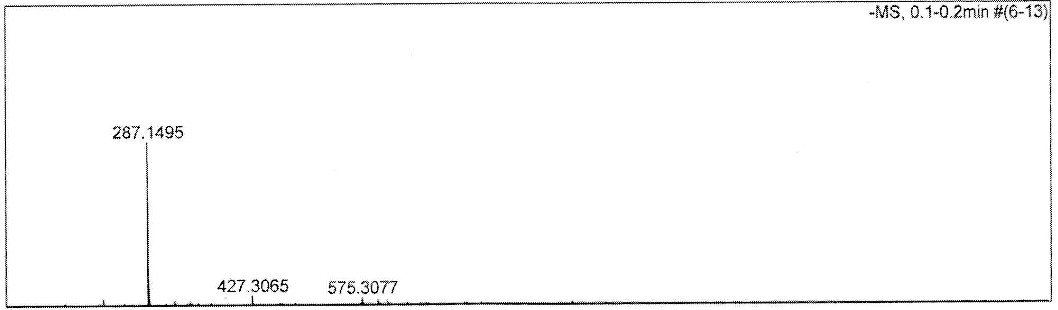


**Figure S6.** HRMS spectrum of 2-*O*-octyl-L-ascorbic acid (**2**)


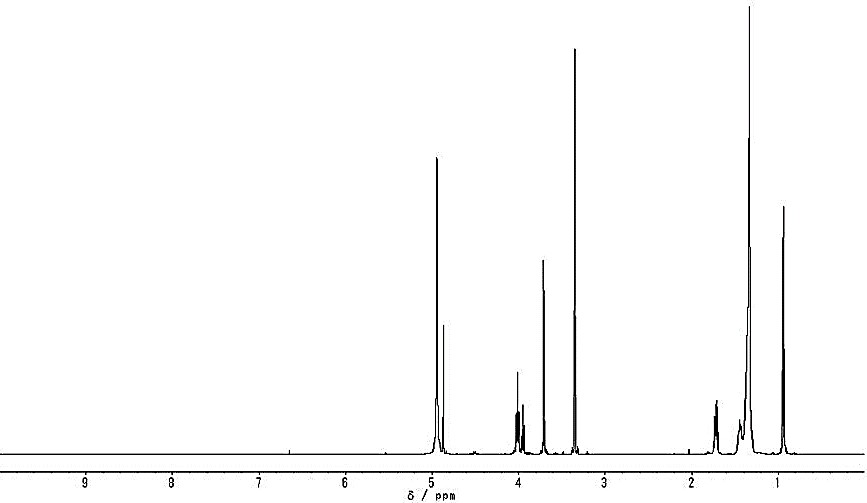


**Figure S7.** ^1^H-NMR spectrum of 2-*O*-dodecyl-L-ascorbic acid (**3**)


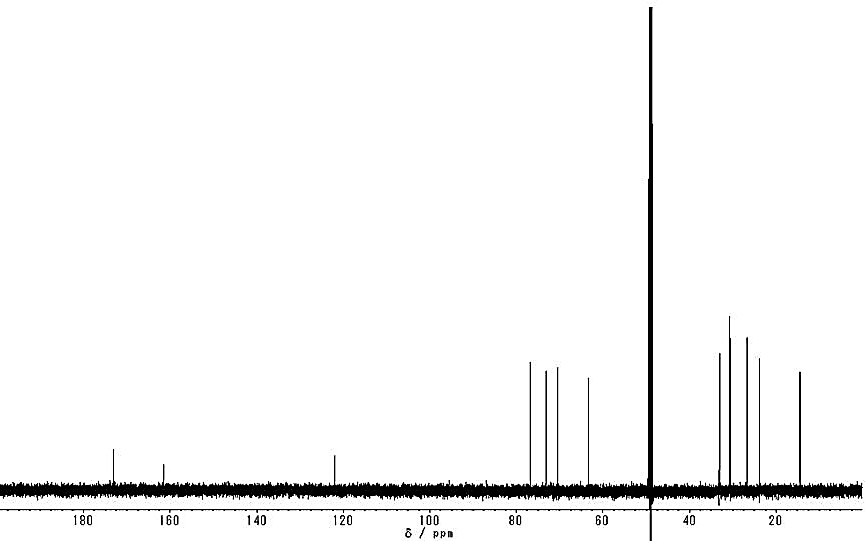


**Figure S8.** ^13^C-NMR spectrum of 2-*O*-dodecyl-L-ascorbic acid (**3**)


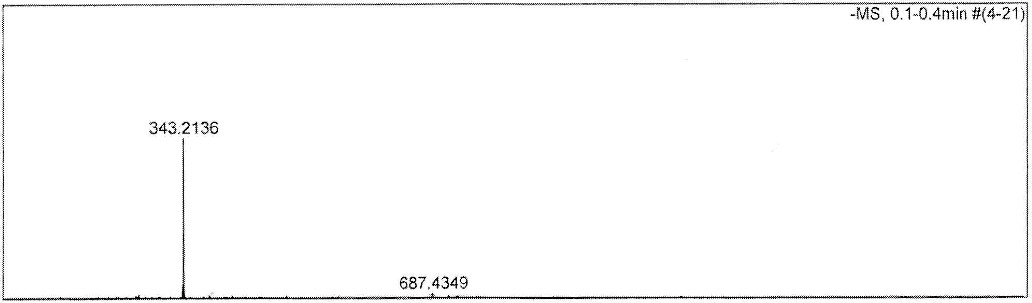


**Figure S9.** HRMS spectrum of 2-*O*-dodecyl-L-ascorbic acid (**3**)


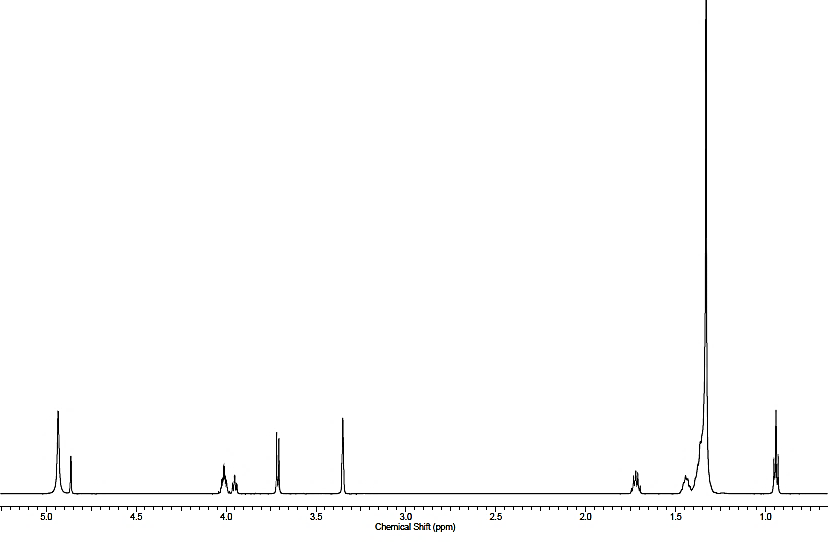


**Figure S10.** ^1^H-NMR spectrum of 2-*O*-hexadecyl-L-ascorbic acid (**4**)


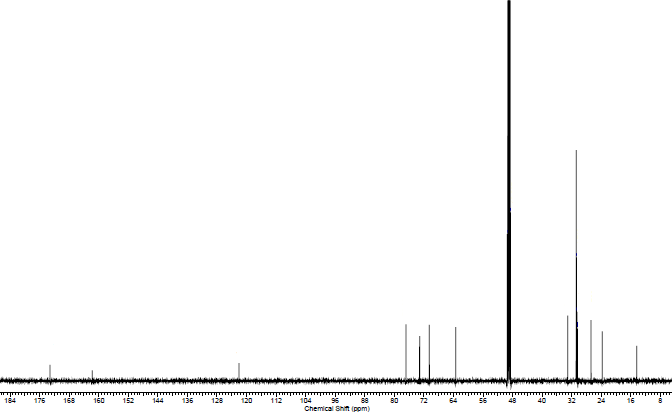


**Figure S11.** ^13^C-NMR spectrum of 2-*O*-hexadecyl-L-ascorbic acid (**4**)


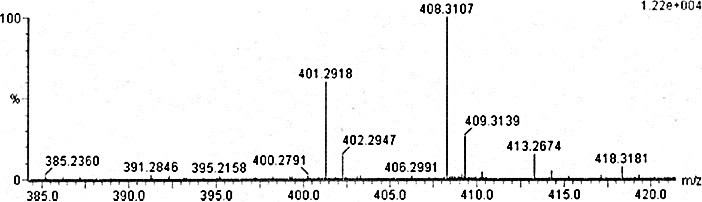


**Figure S12.** HRMS spectrum of 2-*O*-hexadecyl-L-ascorbic acid (**4**)


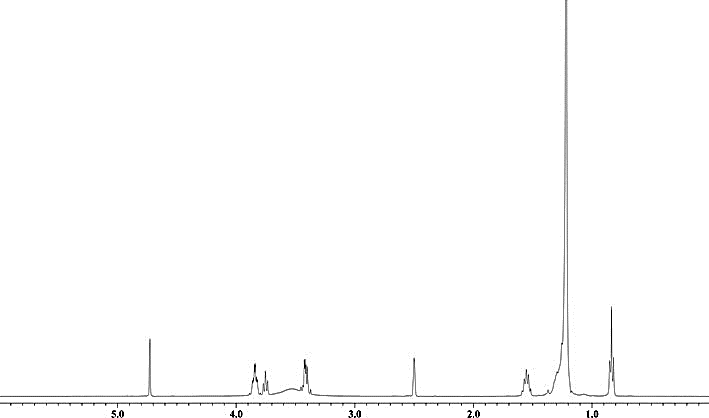


**Figure S13.** ^1^H-NMR spectrum of 2-*O*-octadecyl-L-ascorbic acid (**5**)


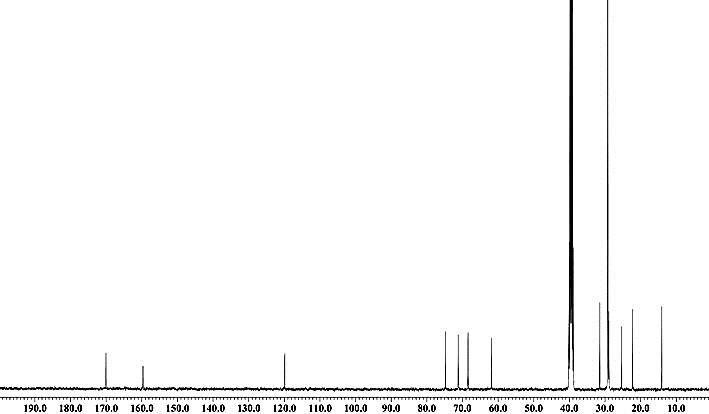


**Figure S14.** ^13^C-NMR spectrum of 2-*O*-octadecyl-L-ascorbic acid (**5**)


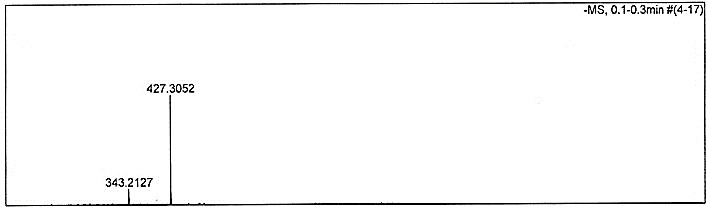


**Figure S15.** HRMS spectrum of 2-*O*-octadecyl-L-ascorbic acid (**5**)


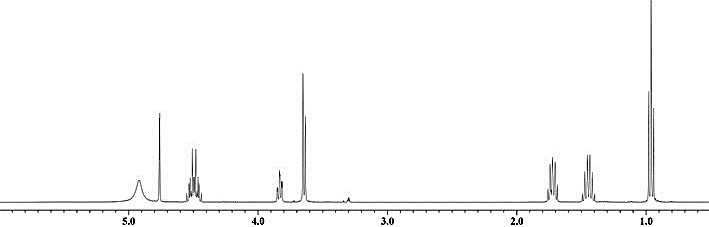


**Figure S16.** ^1^H-NMR spectrum of 3-*O*-butyl-L-ascorbic acid (**6**)


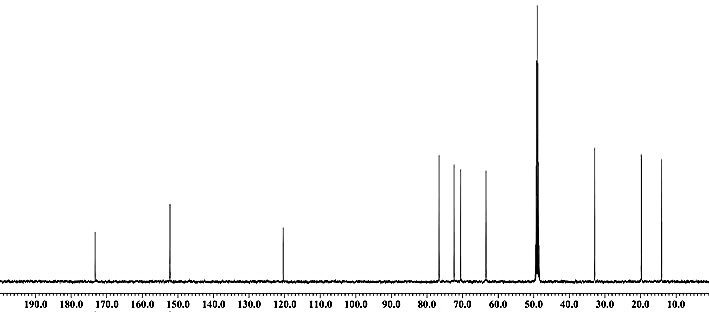


**Figure S17.** ^13^C-NMR spectrum of 3-*O*-butyl-L-ascorbic acid (**6**)


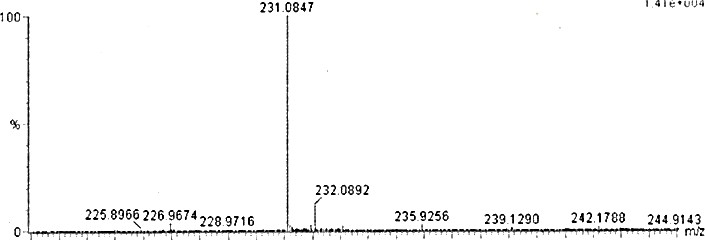


**Figure S18.** HRMS spectrum of 3-*O*-butyl-L-ascorbic acid (**6**)


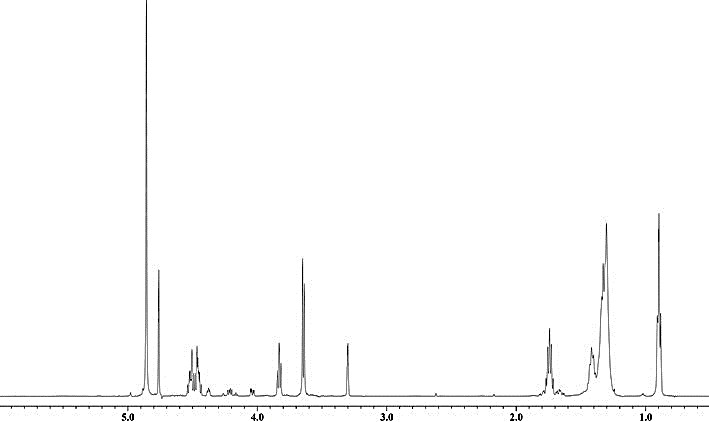


**Figure S19.** ^1^H-NMR spectrum of 3-*O*-octyl-L-ascorbic acid (**7**)


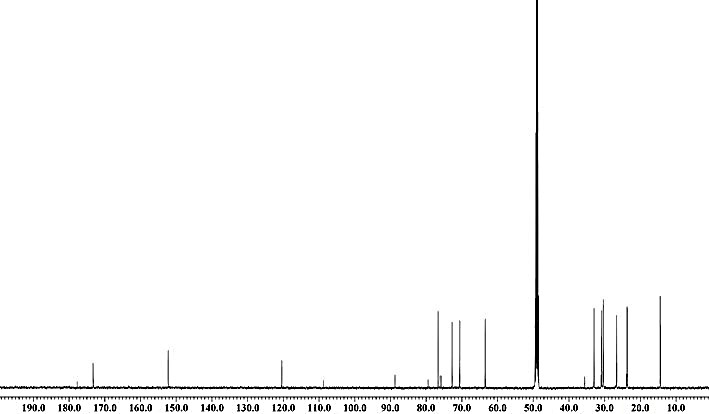


**Figure S20.** ^13^C-NMR spectrum of 3-*O*-octyl-L-ascorbic acid (**7**)


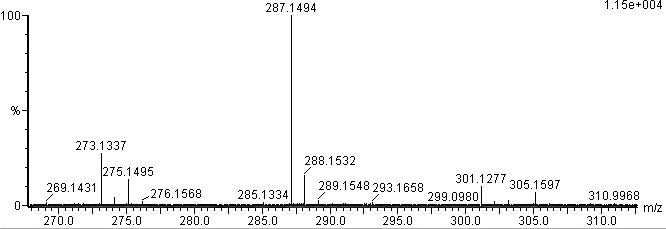


**Figure S21.** HRMS spectrum of 3-*O*-octyl-L-ascorbic acid (**7**)


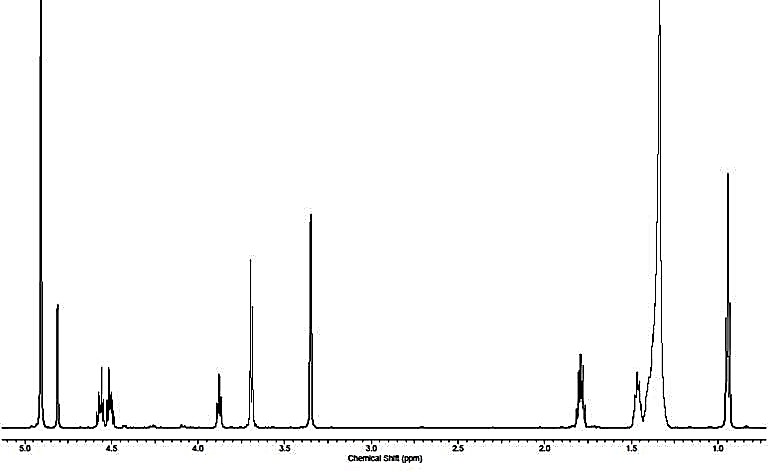


**Figure S22.** ^1^H-NMR spectrum of 3-*O*-dodecyl-L-ascorbic acid (**8**)


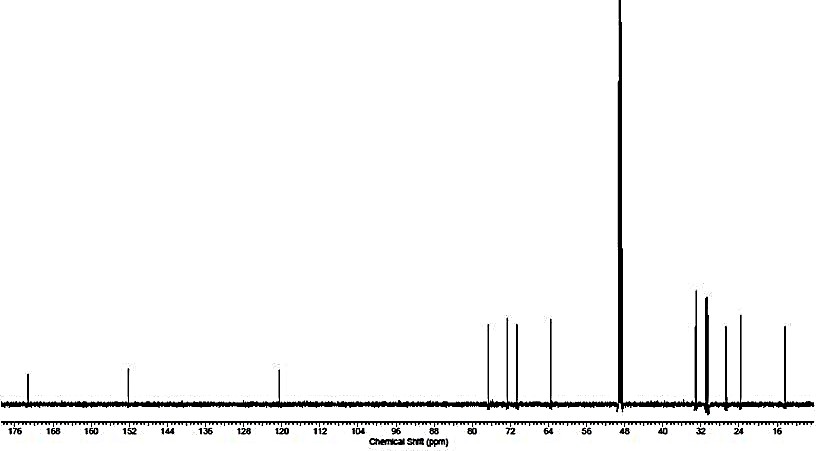


**Figure S23.** ^13^C-NMR spectrum of 3-*O*-dodecyl-L-ascorbic acid (**8**)


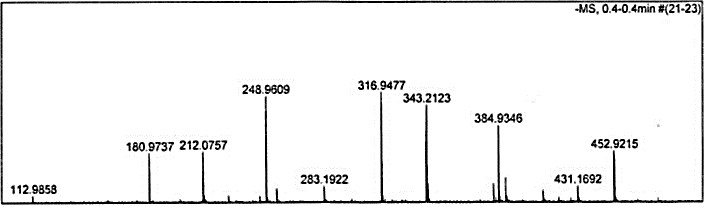


**Figure S24.** HRMS spectrum of 3-*O*-dodecyl-L-ascorbic acid (**8**)


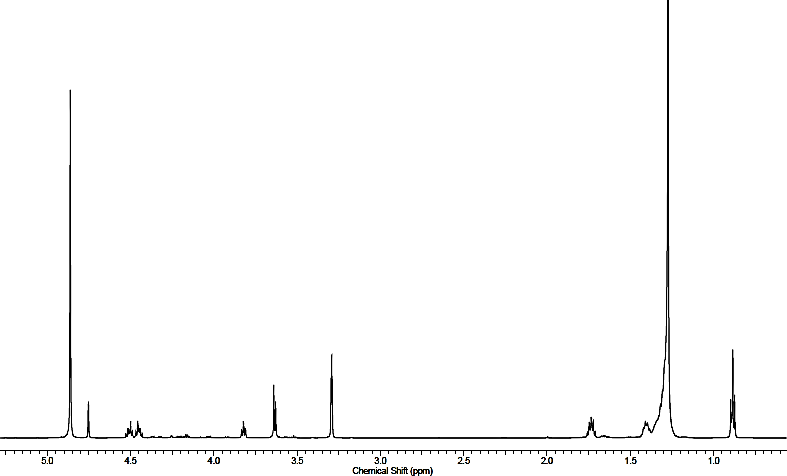


**Figure S25.** ^1^H-NMR spectrum of 3-*O*-hexadecyl-L-ascorbic acid (**9**)


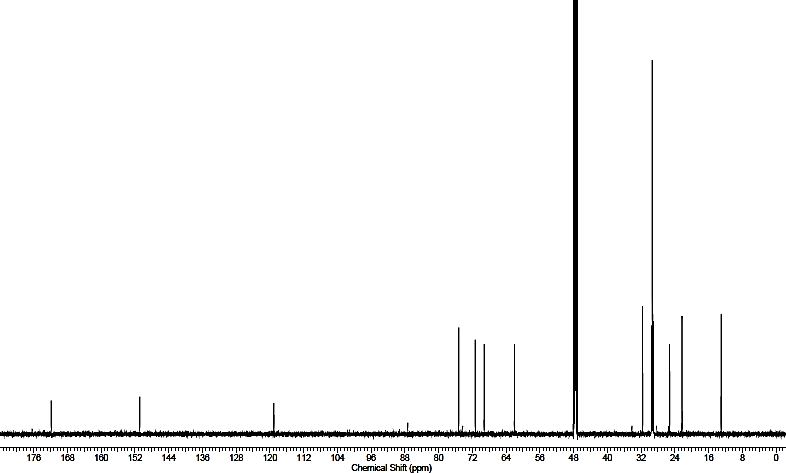


**Figure S26.** ^13^C-NMR spectrum of 3-*O*-hexadecyl-L-ascorbic acid (**9**)


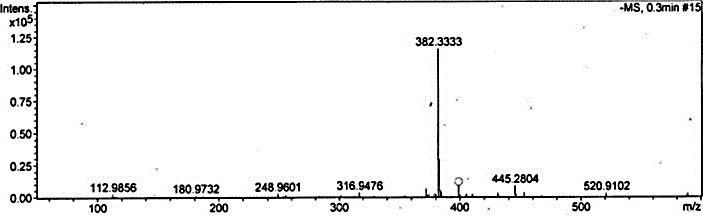


**Figure S27.** HRMS spectrum of 3-*O*-hexadecyl-L-ascorbic acid (**9**)


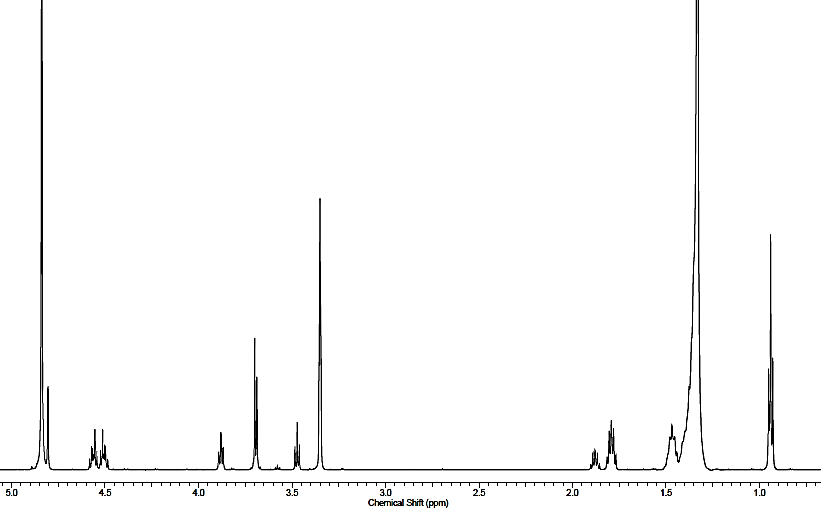


**Figure S28.** ^1^H-NMR spectrum of 3-*O*-octadecyl-L-ascorbic acid (**10**)


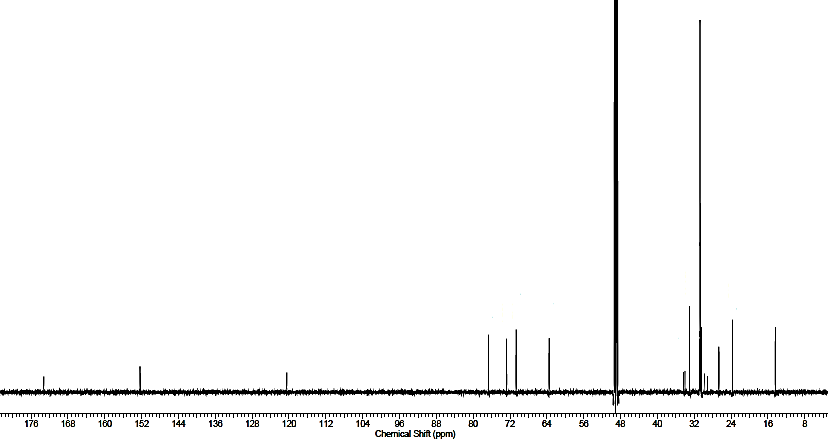


**Figure S29.** ^13^C-NMR spectrum of 3-*O*-octadecyl-L-ascorbic acid (**10**)


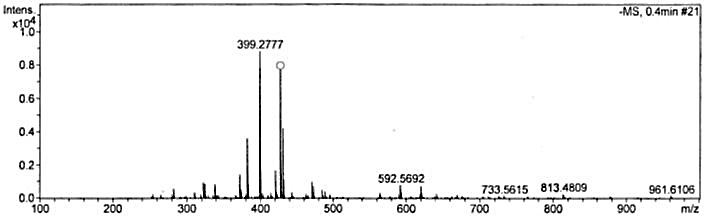


**Figure S30.** HRMS spectrum of 3-*O*-octadecyl-L-ascorbic acid (**10**)


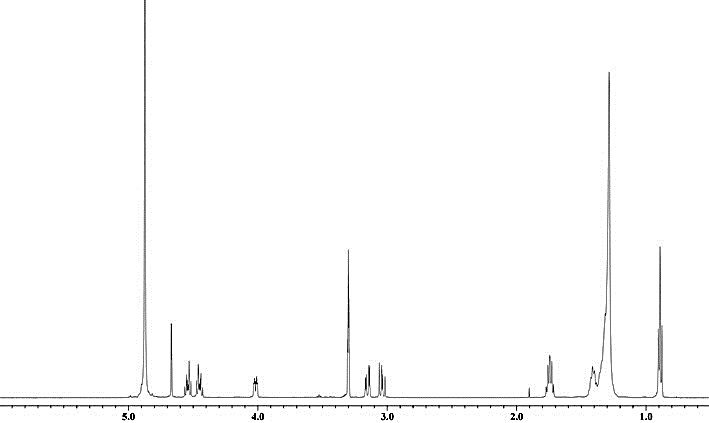


**Figure S31.** ^1^H-NMR spectrum of 6-deoxy-6-amino-3-*O*-dodecyl-L-ascorbic acid (**11**)


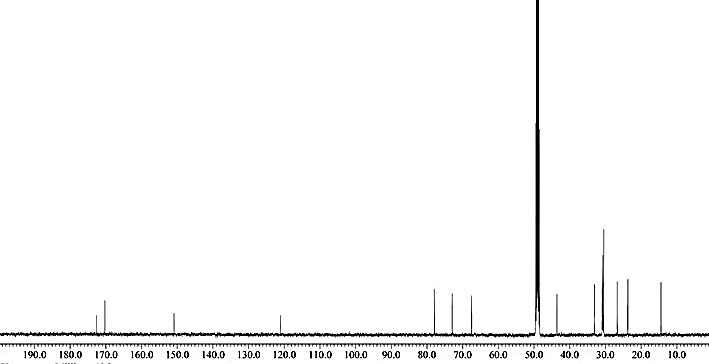


# **Figure S32.** ^13^C-NMR spectrum of 6-deoxy-6-amino-3-*O*-dodecyl-L-ascorbic acid (**11**)


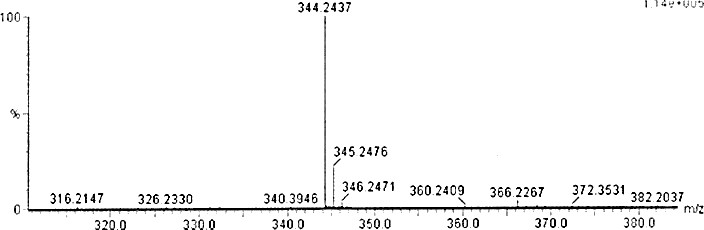


**Figure S33.** HRMS spectrum of 6-deoxy-6-amino-3-*O*-dodecyl-L-ascorbic acid (**11**)


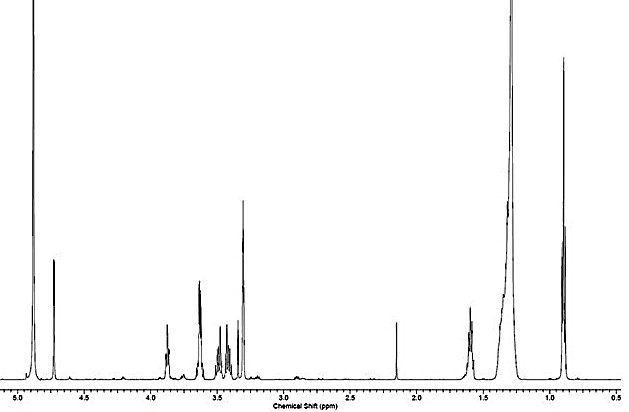


**Figure S34.** ^1^H-NMR spectrum of 3-deoxy-3-dodecylamino-L-ascorbic acid (**12**)


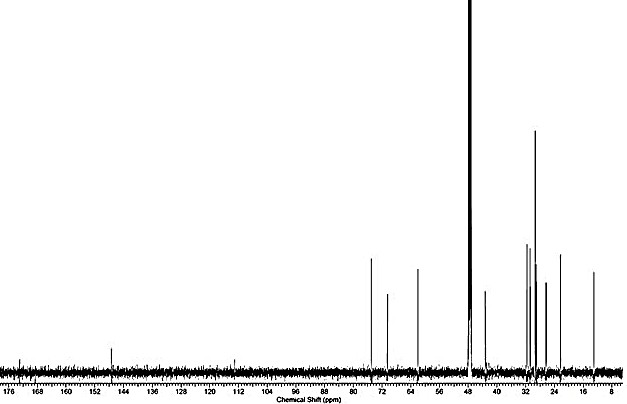


# **Figure S35.** ^13^C-NMR spectrum of 3-deoxy-3-dodecylamino-L-ascorbic acid (**12**)


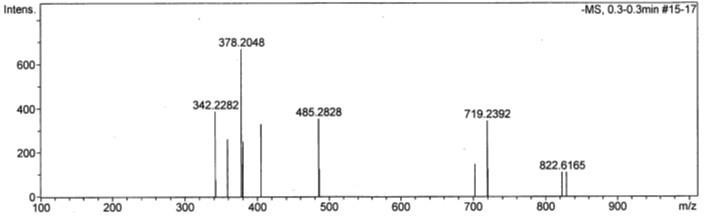


**Figure S36.** HRMS spectrum of 3-deoxy-3-dodecylamino-L-ascorbic acid (**12**)
